# Delayed Gut Colonization Changes Future Insulin Resistance and Hepatic Gene Expression but Not Adiposity in Obese Mice

**DOI:** 10.1155/2024/5846674

**Published:** 2024-09-25

**Authors:** Maria B. B. Ebert, Caroline M. J. Mentzel, Anders Brunse, Lukasz Krych, Camilla H. F. Hansen

**Affiliations:** ^1^ Department of Veterinary and Animal Sciences Faculty of Health and Medical Sciences University of Copenhagen, Frederiksberg, Denmark; ^2^ Department of Food Science Faculty of Science University of Copenhagen, Frederiksberg, Denmark

## Abstract

**Objective:**

The importance of early microbial dysbiosis in later development of obesity and metabolic disorders has been a subject of debate. Here we tested cause and effect in mice.

**Methods:**

Germ-free male Swiss Webster mice were colonized in a specific-pathogen-free (SPF) facility at 1 week (1W) and 3 weeks (3W) of age. They were challenged with a high-fat diet and their responses were compared with SPF mice. Gut microbiota was analyzed by 16S rRNA gene sequencing. Moreover, RNA sequencing of the liver was performed on additional 3W and SPF mice on a regular chow diet.

**Results:**

There were no significant differences in weight, food consumption, epididymal fat weight, HbA1c levels, and serum insulin and leptin, whereas the early germ-free period resulted in mice with impaired glucose tolerance. Both the 1W and 3W group peaked 56% (*p* < 0.05) and 66% (*p* < 0.01) higher in blood glucose than the SPF control group, respectively. This was accompanied by a 45% reduction in the level of the anti-inflammatory cytokine IL-10 in the 1W mice (*p* < 0.05). There were no differences in the gut microbiota between the groups, indicating that all mice colonized fully after the germ-free period. Marked effects on hepatic gene expression (728 differentially expressed genes with adjusted *p* < 0.05 and a fold change ± 1.5) suggested a potential predisposition to a higher risk of developing insulin resistance in the 3W group.

**Conclusions:**

Lack of microbes early in life had no impact on adiposity but led to insulin resistance and altered liver gene expression related to glucose metabolism in mice. The study strongly supports the notion that microbial signaling to the liver in the beginning of life can alter the host's risk of developing metabolic disorder later in life.

## 1. Introduction

A strong consensus has developed around imbalanced gut microbiota, i.e., dysbiosis, as a key player in the pathophysiology of obesity. This was early on supported by the notion that germ-free mice seemed to be resistant to diet-induced obesity [1], but later discordant results indicated that such an observation depends on mouse strain and diet used [2–5]. Several attempts to characterize and define an obese and lean gut microbiota composition were made, and differences have been found on both phylum and genus levels [6–8]. The differences are not the same across studies [9]. Some find, for example, an increase in the Firmicutes : Bacteroidetes ratio [6–8], while others find no changes in the ratio or point to specific taxa or microbial metabolites [10]. Pivotal studies demonstrated that fecal microbiota transplantations from obese and lean mice [11] or human [12] donors to germ-free recipients resulted in body composition phenotypes matching the donors, though the reproducibility was later debated [13], partly due to the technical challenge of successfully transferring a complete human microbiome to mice [14].

Dysbiosis early in life precedes the development of childhood obesity [15], pointing towards a critical window in time during which the microbiome may enable future metabolic responses. Antibiotic treatment in postnatal life increases weight gain both in humans and treated mice [16–20], which persists even after ceased antibiotic treatment. However, in most of those scenarios, the microbiome did not fully recover, questioning the importance of the early life period as the critical window in time as the long-term physiological effects on the host may simply be explained by persistent changes in the microbiome. In addition, some long-term effects of antibiotics on metabolism have been suggested to involve permanent enrichment of antibiotic-resistant or endotoxin synthesis associated genes that may induce low-grade inflammation and insulin resistance and alter production of microbiota metabolites with obesogenic effect [21, 22]. It is also important to note that studies investigating the short and long-term impact of early antibiotic use vary with treatment dose, duration, and class used as well as the experimental study design, which makes it challenging to draw final conclusions. For example, three other independent animal studies found that antibiotic treatment of mice or rats before weaning had no influence on weight gain, especially when controlling for litter size [23–25]. Hence, the relationship between the early microbiome, excessive weight gain, and metabolic dysfunction later in life needs further exploration, and another approach is warranted to clarify the importance of the first intestinal colonizers on host adiposity and metabolism.

## 2. Methods

### 2.1. Ethics

The Animal Experiments Inspectorate under the Ministry of Food, Agriculture, and Fisheries of Denmark approved the study (license ID 2017-15-0201-01262) according to the principles of the European Union Directive 2010/63/EU and the Danish Animal Experimentation Act (No. 474 15/05/2014). The studies were performed in accordance with the Guide for the Care and Use of Laboratory Animals of the National Research Council.

### 2.2. Animals, Housing, and Diet

**Figure 1:**
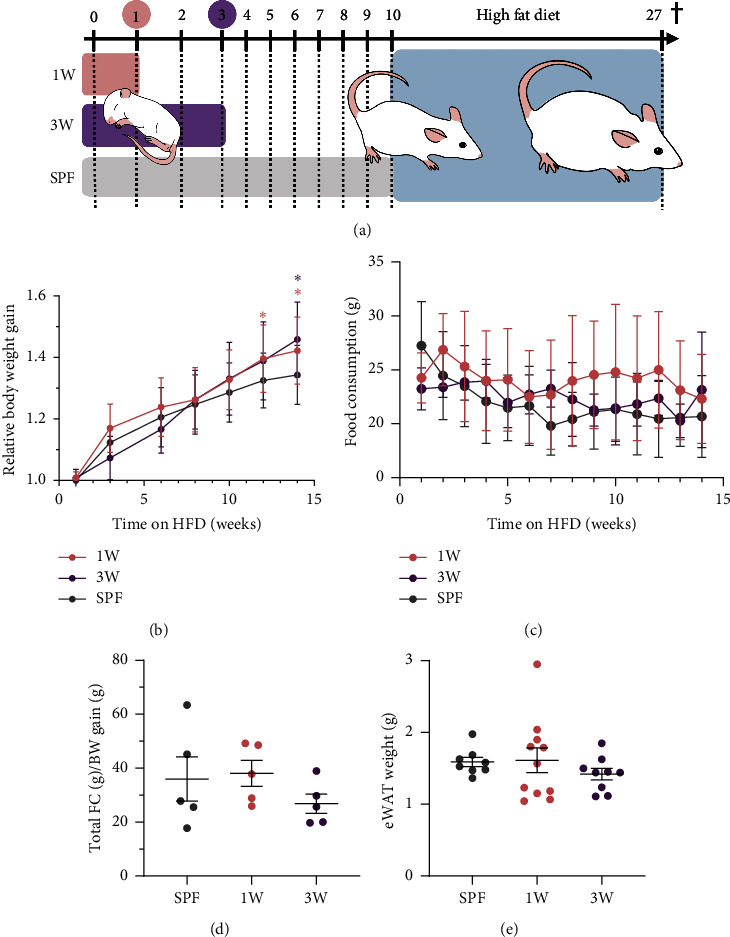
Sensitivity to diet-induced obesity in male SPF and ex-germ-free mice. (a) The experimental setup is outlined with two ex-germ-free groups colonized at one-week-of-age (1W, *n* = 14) or three week-of-age (3W, *n* = 11), and one microbiota-associated SPF bred control group (SPF, *n* = 10). At 10 week-of-age, all mice were put on a high-fat diet for 17 weeks before the mice were euthanized at 27 week-of-age. (b) The weight was measured on all mice during high-fat diet feeding and the average relative weight gain is shown. Data points from mice that started losing weight because of diabetes were excluded. Error bars represent SD. (c) The average weekly food consumption of high-fat diet per mouse per cage is shown. Error bars represent SD (*n* = 5-6 cages per group). (d) Food efficiency based on the total average food consumption and total average body weight gain per cage during the 14 weeks high-fat diet feeding period is shown. Mean and SEM are shown. (e) Epididymal white adipose tissue (eWAT) was measured at euthanasia after 17 weeks on high-fat diet. Mean and SEM are shown. ^∗^*p* value below 0.05.

Germ-free Swiss Webster mice (Taconic, Germantown, NY) were housed in our AAALAC accredited germ-free facility (University of Copenhagen, Frederiksberg, Denmark) in HEPA-ventilated isolators (PFI systems, Milton Keynes, UK; pressure 110 pascal, 23°C) with free access to an irradiated Altromin 1314 diet (Brogaarden, Lynge, Denmark) and sterile water. Breeding pairs were transferred a few weeks before mating to our AAALAC accredited barrier protected facility where they were cohoused with SPF mice already in the facility. The facilities maintained a controlled and consistent environment, with a room temperature of 22 ± 2°C, a relative humidity of 45–55%, air changes 15–20 times per hour, and a 12/12 hour light/dark cycle. Health monitoring, following international FELASA guidelines, revealed no indications of listed infections (SPF). Their male pups (C, *n* = 10) were used as control mice that were fully colonized throughout life. Germ-free male pups were transferred to the barrier facility one week (1W, *n* = 14) or three weeks (3W, *n* = 11) after birth together with the dams of the preweaned pups and were given dirty bedding from the cages containing the barrier-bred control mice to allow a complete conventionalization of the microbiome (indifferent from the SPF control mice). Male pups from at least three random litters were included in each group. The primary read outs weight and glucose tolerance were used to determine group sizes in an 80% power calculation (min 10 males/group, final number depended on the number of males in each litter). The mice were postweaning housed in open cages with littermates in groups ranging from two to three mice per cage that were blinded to the researchers and fed the same diet as in the isolators until 10 weeks of age before they switched to a high-fat diet for 17 weeks (Figure 1(a)). The high-fat diet had an energy density of 5.21 kcal/g (fat 60% kcal, carbohydrate 20% kcal, and protein 20% kcal) (Research Diets, New Brunswick, New Jersey). The food was weighed and changed for each cage two times a week and the mice were weighed every second week for 14 weeks until the more stressful *in vivo* procedures started that may affect the weight.

### 2.3. Diabetes

Mice were observed for polyuria and polydipsia weekly at cage change. Glycated hemoglobin (HbA1c) was measured on nonfasted tail blood using a DCA Vantage Analyzer (Siemens Healthcare Diagnostics, Ballerup, Denmark) on animals from extra wet cages or if they started to lose weight. Mice were diagnosed with diabetes and euthanized if HbA1c levels were above 6.5%. A total of 9 mice were diagnosed after 15 weeks on high-fat diet and excluded from further analyses, as diabetes results in weight loss, heightened inflammatory markers, and increased glucose intolerance, that would bias the results on the primary read outs.

### 2.4. Glucose Tolerance, Hyperglycemia, and Insulinemia

After 15 weeks on high-fat diet, an oral glucose tolerance test was performed. The mice were fasted 6 hours prior to the test, and the fasting blood glucose level (baseline) was measured with a glucometer, using a drop of blood from a lateral tail vein puncture. After administering a fixed dose of 75 mg glucose monohydrate (500 mg/ml) by oral gavage at time 0, the blood glucose level was measured with a glucometer after 15, 30, 60, 90, and 120 minutes. At euthanization, glycated hemoglobin (HbA1c) was measured on nonfasted tail blood using a DCA Vantage Analyzer (Siemens Healthcare Diagnostics, Ballerup, Denmark). Mice were then fasted for 6 hours, blood glucose was measured with a glucometer, and mice were sedated with a Hypnorm (0.315 mg/ml fentanyl citrate and 10 mg/ml fluanisone, Vetapharma, Leeds, United Kingdom) and Dormicum (5 mg/ml, B. Braun, Melsungen, Germany) mixture for eye blood collection before cervical dislocation.

### 2.5. Serum Multiplex Analysis

Serum insulin and leptin levels were measured with the Mouse Metabolic kit (Meso Scale Discovery (MSD), Rockville, MD) according to manufacturer's protocol. Serum concentrations of cytokines IFN-*γ*, IL-1*β*, IL-2, IL-4, IL-5, IL-6, IL-9, IL-10, IL-12p70, IL-15, TNF-*α*, IL-17 A/F, IL-30, and IL-33 and chemokines CXCL-1, CXCL-2, CXCL-10, CCL-2, and CCL3 were measured using V-PLEX Mouse Cytokine 19-Plex Kit (MSD) on MESO QuickPlex SQ120 and analyzed by the standard software Discovery Workbench v4 (MSD).

### 2.6. Gut Microbiota Analysis

Germ-free status was tested both by aerobic and anaerobic culturing on agar plates and PCR methods [26]. DNA was extracted with QIAamp DNA stool mini kit (Qiagen, Hilden, Germany). The V3-V4 region of the 16S rRNA gene was amplified using the following primers (NXt_V3-V4_F 59-TCGTCGGCAGCGTCAGATGTGTATA-AGAGACAGCCTAYGGGRBGCASCAG-39 and NXt_V3-V-4_R 59-GTCTCGTGGGCTCGGAGATGTGTATAAGAGA-CAGGGACTACNNGGGTATCTAAT-39). PCR reactions containing 12 ml AccuPrime SuperMix II (Thermo Fisher Scientific), 0.5 ml of each primer (10 mM), 5 ml of genomic DNA (20 ng/ul), and nuclease-free water to a total volume of 20 ml were run on SureCycler 8800 (Agilent, Santa Clara, CA). Cycling conditions applied were as follows: denaturation at 95°C for 2 min; 33 cycles of 95°C for 15 s, 55°C for 15s, and 68°C for 40 s, followed by final elongation at 68°C for 5 min.

The fecal microbiota composition was determined for the mice at 10 weeks of age before the high-fat diet was given to ensure that the ex-germ-free mice had colonized similarly to the SPF control mice, only at a later time point. The relative abundance of the bacterial community was evaluated using nanopore-based sequencing of the near full 16S rRNA gene amplicon using GridIONx5 (Oxford Nanopore Technologies, Oxford, UK) as previously described [27]. The generated abundance table was further analyzed using the Qiime2 (v2018.11) bioinformatic platform. Alpha diversity (observed species index) and beta diversity (PCoA based on Bray–Curtis and Jaccard distance matrices) were calculated using rarefaction to 13,000 reads per sample. Permutational multivariate analysis of variance (PERMANOVA) was used to test differences between the three groups based on Bray–Curtis and Jaccard distance matrices.

### 2.7. Liver Cytokines

A 1/2-1 cm piece of the liver lobus medialis was homogenized in 400 *μ*l lysis buffer (Tris lysis solution with 1 : 100 phosphatase inhibitor 1 and 2, and 1 : 100 protease inhibitor from the MSD inhibitor pack, Meso Scale Discovery). Samples were diluted 1 : 2 and analyzed for IFN-*γ*, IL-1*β*, IL-2, IL-4, IL-5, IL-6, IL-10, IL-12p70, KC/GRO, and TNF-*α* with V-PLEX Proinflammatory Panel 1 Mouse kit (Meso Scale Discovery) similarly as described for the serum cytokines. IL-10 was the only cytokine below detection level. The concentrations were normalized to total protein measured with Pierce Detergent Compatible Bradford Assay kit according to manufacturer's protocol.

### 2.8. Liver Gene Expression

**Figure 2:**
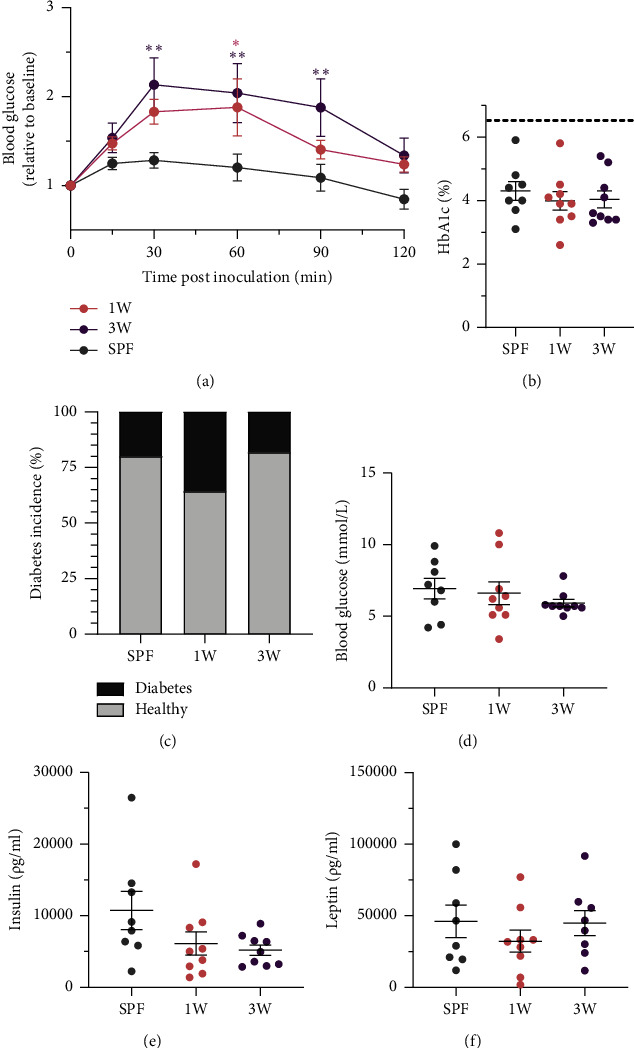
Oral glucose tolerance, hormone levels, and diabetes in male SPF and ex-germ-free mice. (a) The oral glucose tolerance after 15 weeks on high-fat diet in SPF mice (*n* = 8) and mice that were germ-free until 1W (*n* = 9) or 3W (*n* = 9) is shown. Blood glucose levels were measured at 15, 30, 60, 90, and 120 min post oral glucose of a fixed dose of glucose and the relative levels compared to baseline is shown. (b) Blood HbA1c levels are shown for the nondiabetic mice at euthanasia after 17 weeks on high-fat diet. The dotted line represents the cutoff value for diagnosing diabetes. (c) The diabetes incidence during the 17 weeks on high-fat diet is shown for the SPF (*n* = 2 out of 10 mice), 1W (*n* = 5 out of 14 mice), and 3W (*n* = 2 out of 11 mice) groups. (d) Fasting blood glucose, (e) insulin, and (f) leptin levels at euthanasia after 17 weeks on high-fat diet are shown. Mean and SEM are shown in all plots. ^∗^*p* value below 0.05 and ^∗∗^*p* value below 0.01.

Additional groups of male Swiss Webster mice that remained on the low-fat breeding diet (Altromin 1314 diet) were colonized as SPF mice throughout life (SPF, *n* = *5*) or at 3 weeks of age (3W, *n* = *5*) and killed at 7 weeks age. The liver lobus dexter was sampled in RNA later and stored at −80°C. RNA was purified using the RNeasy Lipid kit (Qiagen, Hilden, Germany), and RNA integrity was measured on a Bioanalyzer (Agilent Technologies, Santa Clara, CA) and samples below a RIN score of 7 were abolished for further processing. Total RNA was sent to the commercial sequencing service at Novogene (Beijing, China) for RNA-seq library preparation, Illumina sequencing, and bioinformatic processing of the raw reads. Differential expression analysis of the 31031 expressed genes by DESeq2 yielded a total of 738 differentially expressed genes, where 407 were upregulated and 321 downregulated (adjusted *p* value <0.05 and fold change ± 1.5) [28]. The PCA plot was generated using DEseq2 and the volcano plot was generated using EnhancedVolcano as previously described [29]. The Metascape webserver (https://www.nature.com/articles/s41467-019-09234-6#citeas) was used for gene-enrichment analysis of the differentially expressed genes. The gene enrichment analysis output is provided in Supplementary Table 1 and Figure 2. The enrichment analysis output was queried for the keywords “insulin,” “glucose,” and “carbohydrate” to extract core genes relevant for the phenotype that was present in at least two pathways. 22 core genes were produced using the DESeq2 output and GGplot2 in R.

### 2.9. Liver Histology

Formalin-fixed lobus dexter of the liver was paraffin-embedded, cut in 5 micrometer sections on a sliding microtome, and stained with hematoxylin and eosin for histological examination. 8-bit RGB color images were captured at 2.5x magnification on a Leica DM 2500 microscope using an MC 190 HD camera (both Leica Microsystems, Bronshoj, DK). Images were imported to Fiji image analysis software, and steatotic areas were assigned with the free-hand selection tool by a blinded researcher. The total steatotic area was estimated using ROI manager and expressed relative to the total tissue area.

### 2.10. Statistics

GraphPad Prism version 9 (GraphPad Software, San Diego, CA) was used for statistical analysis and *p* values less than 0.05 were considered significant. Gaussian distribution tests (D'Agostino and Pearson omnibus, or Shapiro–Wilk normality test if *n* < 5) were applied to all quantitative data. Data were analyzed using one-way ANOVA with Dunnett's multiple comparison test comparing the mean of the ex-germ-free groups to the control group, or nonparametric Kruskal–Wallis test with Dunn's multiple comparison test for data that did not assume Gaussian distribution. Welch's correction was included if variances were unequal by Brown–Forsythe test. Data generated across multiple time points (weight, food consumption, and glucose tolerance test) were analyzed with repeated measures ANOVA. Dichotomous data (diabetes incidence) were analyzed with the chi-square test (*χ*^2^-test).

## 3. Results

### 3.1. Postnatal Germ-Free Life Had No Impact on Later Life Adiposity or Food Consumption

To investigate the impact of the first intestinal colonizers on the host's long-term response to high-fat diet, germ-free mice were colonized at one week-of-age (1W) or three week-of-age (3W) at weaning and put on a high-fat diet later in life. After 14 weeks on high-fat diet, before the more stressful *in vivo* tests began, mice colonized at 1W and 3W had gained more weight compared to their baseline weight compared to the SPF mice that had been colonized their entire life (Figure 1(b)). However, germ-free mice usually weigh more than SPF mice, and on the actual weight data, there were no significant difference between the groups (Figure S1A). In addition, the weight of the epididymal white adipose, measured at termination after 17 weeks on a high-fat diet, was also similar between the three groups (Figure 1(e)). Hence, the ex-germ-free mice gained more weight to reach their expected weight after 14 weeks on high-fat diet, but the delayed colonization had no impact on adiposity. Food consumption was measured per cage during the first 14 weeks of high-fat diet feeding, but the relative weight gain in the ex-germ-free groups was not accompanied by increased food consumption (Figure 1(c)). The calculated feed efficiency was also similar for all three groups (Figure 1(d)).

### 3.2. Delayed Colonization Enhanced Glucose Intolerance

Despite the lack of effect on adiposity, a germ-free period in the beginning of life had a strong impact on the glucose tolerance test performed after 15 weeks on high-fat diet. Both the 1W and 3W groups had a worse glucose tolerance compared to the SPF mice colonized throughout life, the 3W group more so than the 1W group (Figure 2(a)). Despite the increased glucose intolerance, the mice had the same level of long-term blood glucose, HbA1c (Figure 2(b)), and there were no significant differences in the incidence of mice becoming diabetic between the three groups (Figure 2(c)). Fasting blood glucose (Figure 2(d)) and insulin levels (Figure 2(e)) showed a similar trend indicating that insulin resistance rather than insulin production was affected by the delayed colonization. Leptin levels of nondiabetic mice were also not significantly different between the groups (Figure 2(f)), which correlated well with the related epididymal white adipose tissue weight.

### 3.3. High-Fat Diet-Induced Inflammation Was Unaffected by Colonization Pattern

**Figure 3:**
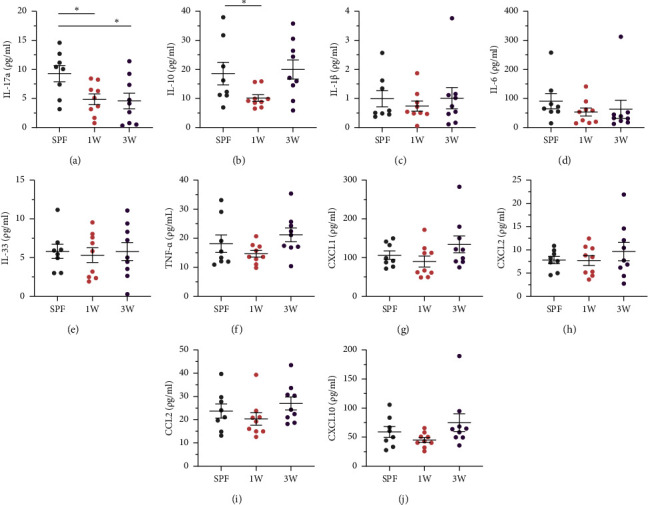
Systemic low-grade inflammation in male SPF and ex-germ-free mice on high-fat diet. (a–j) serum cytokine and chemokine levels for nondiabetic SPF (*n* = 8), 1W (*n* = 9), and 3W (*n* = 9) mice were measured with mesoscale multiplex technology, and the analytes above detection range are shown as indicated. For the shown analytes, measurements below detection range were given the value of half of the lower detection limit as instructed by the manufacturer. Mean and SEM are shown in all plots. ^∗^*p* value below 0.05.

Several cytokines and chemokines were measured in the serum of the nondiabetic mice on the day of euthanization, as we have previously observed that the levels correlate with glucose intolerance in mice on high-fat diet [30]. None of the measured analytes were upregulated in the delayed colonization groups compared to the SPF group (Figures 3(a), 3(b), 3(c), 3(d), 3(e), 3(f), 3(g), 3(h), 3(i), and 3(j)); however, IL-17 was significantly downregulated in both the 1W and 3W groups compared to the SPF group (Figure 3(a)), and the anti-inflammatory cytokine IL-10 was reduced only in the 1W group (Figure 3(b)).

### 3.4. Delayed Colonization Did Not Alter the Adult Microbiome Composition

To investigate whether the host response was in fact determined by delayed colonization or different microbiome compositions post colonization, 16S sequencing of fecal pellets sampled before starting the high-fat diet was performed. Principal coordinate analysis (PCoA) plot based on Jaccard and Bray–Curtis distance matrices showed that the ex-germ-free groups clustered together with the SPF group (Figure S1B–C). Hence, the mice were considered fully colonized after conventionalization, and any differences between the groups were, thus, solely due to the early germ-free period.

### 3.5. The Early Microbiome Has Long-Term Effects on Hepatic Gene Expression

**Figure 4:**
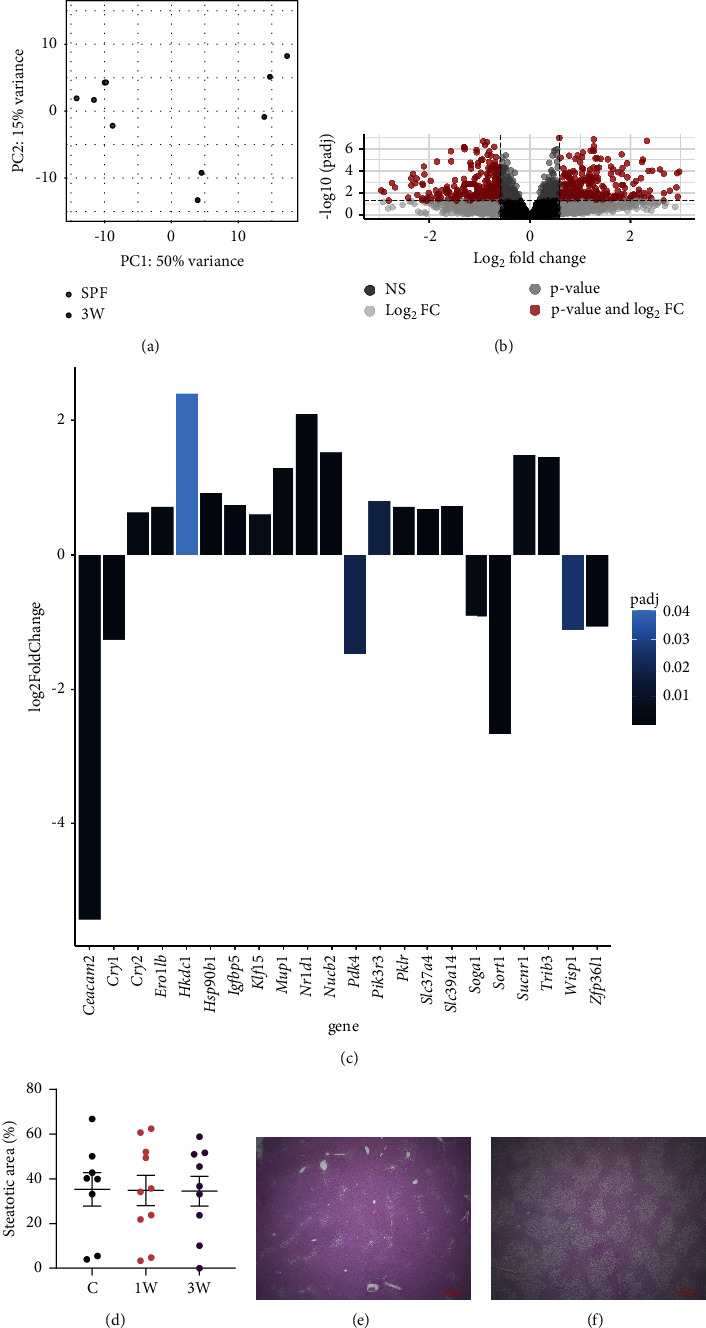
Liver gene expression and steatosis in male SPF and ex-germ-free mice. (a) Principal component analysis plot of liver RNA-seq data from SPF (*n* = 5) and ex-germ-free (ex-GF, *n* = 5) male mice that were colonized at 3 weeks of age. (b) Volcano plot of differentially expressed genes in liver tissue with a total of 31031 expressed genes. Red dots represent a total of 728 genes above the significance threshold with an adjusted *p* value <0.05 (−log_10_ (p.adj) > 1.3), and above the fold change (FC) threshold of 1.5x higher or lower than the SPF group (Log_2_ FC > 0.585 or < −0.585). (c) Bar plot of the log_2_ fold change and adjusted *p* value of 22 core insulin and glucose related genes. (d) Histological analysis of the steatotic area in the liver of male nondiabetic SPF (*n* = 8), 1W (*n* = 10), and 3W (*n* = 9) mice after 10 weeks on high-fat diet. Representative images of H&E stained liver sections from a mouse with low (e) and high (f) degree of steatosis are shown.

Insulin resistance is a pathophysiological hallmark of progressive liver disease [31]. Therefore, we aimed to investigate whether the high glucose intolerance in mice that were germ-free for the first 3 weeks of their life also led to altered hepatic function and/or inflammation. Principal component analysis of RNA sequencing data revealed that the SPF and 3W groups clustered significantly different (Figure 4(a)). The volcano plot indicated significant differential expression of 728 genes out of a total of 31,031 expressed genes at 7 weeks of age, attributed to the germ-free preweaning period, based on the set threshold (Figure 4(b) and Supplementary Table S1). Gene enrichment analysis revealed that the top gene ontology pathways of the regulated genes were enriched in very general regulatory process pathways (Figure S2A) and showed a liver specific expression pattern (Figure S2B). However, when querying the significant enrichment analysis output (Supplementary Table S1) for insulin, glucose, and carbohydrate-related pathways, a total of 22 core genes present in at least two pathways were identified (Figure 4(c)). In support, diabetes mellitus was also one of the identified pathways in the enrichment analysis when mapped to human genes (Figure S2C).

Cytokine measurements in the liver of the 1W, 3W, and SPF mice after 10 weeks on a high-fat diet verified the lack of effect on liver inflammation. Cytokine levels were similar between the three groups, except for reduced IL-6 concentration in the liver of 3W mice compared to the SPF mice (Figure S3). In addition, histological examination of the liver at the same time point showed similar degree of steatosis between the three groups and lack of inflammation and fibrosis in all samples (Figures 4(d), 4(e), and 4(f)). Hence, the liver pathology was unaffected in the high-fat diet-induced obesity model.

## 4. Discussion

When we are born, the first thing to greet us is the bacteria residing in the birth canal and perianal area of the mother. Next in line are the medical staff, hospital equipment, parents, and all the bacteria that come along with them [32]. These bacteria start to colonize every little inch of the internal and external surface of the infant, eventually becoming just as many as, or even outnumber the human cells [33]. The majority of these bacteria reside in the gastrointestinal tract, particularly in the colon. This community, known as the gut microbiome, has gained tremendous attention in the last two decades, with studies finding associations between alterations in the early or established gut microbiota composition and a wide range of health issues, among these obesity and diabetes [34–36]. However, it is not clear whether the first intestinal colonizers play a long-term causative role for the risk of becoming overweight and insulin resistant. Therefore, this study investigated the development of obesity and diabetes in high-fat diet fed mice that were germ-free for only a short period after birth, to clarify the role of the very first colonizers of the gut. We found that the absence of the gut microbiota in the early weeks plays no role in regulating weight gain later in life, as no differences were observed in the body weight between the groups. The only exception was a lower body weight in the germ-free mice at weaning, just before transfer to the barrier-bred facility, which indicates that the mice were not prone to become more obese on a high-fat diet, but rather caught up on their expected adult weight. This notion was supported by similar findings in eWAT weight and serum leptin levels between the groups. Germ-free and antibiotic treated mice may gain less fat than conventional mice, even on a high-fat diet [1, 16, 37]. However, studies also show that these traits disappear when germ-free mice are conventionalized in adult life [1]. Collectively, these results show that the early life gut microbiota has no role in the host's risk of gaining excessive weight later in life. It is of course important to note that long-term changes in the offspring microbiome due to, for example, maternal diet and health status, antibiotic treatment, etc. may still play a role in the risk of growing up overweight.

In contrast to the above findings, both the glucose tolerance and hepatic function were altered in the late colonized mice, strongly suggesting that the metabolic response to high-fat diet, rather than adiposity, is regulated by the first intestinal colonizers. Previous studies have similarly shown that the microbiota seems to have less impact on diet-induced obesity compared to metabolic responses. Facility-dependent differences in insulin response, for example, are not accompanied by differences in high-fat diet-induced obesity [38]. Furthermore, conventional mice with a diverse microbiota were shown to be more prone to develop metabolic endotoxemia and adipose tissue inflammation in response to a high-fat diet compared to SPF mice, whereas the weight gain was similar [39]. In yet another study, gut microbiota transfer from obese donors to germ-free recipients resulted in transfer of glucose intolerance even though obesity was not transferred [13], suggesting a direct link between microbial dysbiosis and metabolic dysfunction that may to a certain degree be independent of adiposity.

Surprisingly, several of the mice in all groups developed diabetes after 15 weeks on high-fat diet, which is not commonly observed in the frequently used high-fat diet-induced obesity model in C57BL/6 mice. There are known strain differences in the response to high-fat diet, but C57BL/6 mice are usually considered the most sensitive mouse strain related to weight gain [40]. However, another study using Swiss Webster mice also encountered mice with diabetes [41]. Therefore, it seems to be worth considering using this strain in future studies on type 2 diabetes. It is also worth noting that we started the high-fat diet at a late time point at 10 weeks of age. In line with this, adult C57BL/6J mice have previously been shown to be more sensitive to develop obesity and related complications compared to young newly weaned mice traditionally used for the high-fat diet-induced obese model [42]. Further studies with larger group sizes would be of interest to assess the diabetes incidence in different conditions.

A critical window in time exists where gut microbial colonization is necessary for the developing immune system. Delayed colonization in postnatal life of germ-free mice induces long-term changes in various immune cell subsets [43, 44]. In our study, the serum cytokines and chemokines were unaltered by delayed colonization, except for IL-17. Induction of Th17 cells is dependent on the presence of the gut commensal segmented filamentous bacteria [45], which is more abundant early in life and remains low in adulthood [46]. It can therefore be speculated whether segmented filamentous bacteria were present in the beginning of life in the SPF mice, despite their absence at 10 weeks of age, and therefore only induced a Th17 response in those mice in contrast to the 1W and 3W mice where SFB was absent.

While systemic and liver inflammation and pathology was unaffected in the high-fat feeding study, the delayed colonization process significantly changed hepatic gene expression in young animals not subjected to high-fat diet. The most upregulated core genes in the 3W animals included *Nr1d1*, *Sucnr1*, and *Trib3*. Interestingly, *Nr1d1*, a nuclear receptor REV-ERB-*α*, is involved in molecular clock mechanisms and has been linked to metabolism, obesity, and diabetes [47]. *Sucnr1* is involved in glucose homeostasis and is overexpressed in livers of induced NASH mice and protects against further liver injury [48]. Moreover, *Trib3* expression is involved in hepatic insulin resistance in human subjects [49]. Of the most downregulated core genes in the 3W animals, *Ceacam2* and *Sort1* are noteworthy. *Ceacam2*-deficient mice develop insulin resistance [50], and *Sort1* is involved in intracellular glucose transport and its expression is downregulated in diabetic mice and humans [51]. Our study is thus in concordance with other studies demonstrating a link between early life dysbiosis due to, e.g., antibiotic treatment and high-fat diet-induced metabolic aberrations, and verifies the importance of especially the very first colonizers. Our study further suggests that this could be mediated through a gut-liver crosstalk in the very beginning of life, especially considering that microbiota-regulated abnormal liver metabolism in mice has been shown to precede the onset of insulin resistance [52]. However, further studies on the functional importance of the differentially regulated liver genes in microbial regulation of insulin resistance are needed to establish causality.

## 5. Conclusions

The early life gut microbiota has no role in future sensitivity to diet-induced obesity in mice, whereas glucose intolerance and liver expression of metabolism-related genes are significantly altered when the neonatal colonization process of the gastrointestinal tract is delayed. The findings encourage microbiota-mediated intervention to start already during the colonization process of the newborns to reduce the risk of insulin resistance in babies at risk. This could, for example, be relevant in situations where even just a temporary dysbiosis is a risk, such as delivery by cesarean section or postnatal antibiotic treatment.

## Data Availability

RNAseq data are available in NCBI GEO (GSE249397) and 16S sequencing data are available in BioProject ID: PRJNA1048180.

## Supplementary Materials

Supplementary Materials Figure S1: body weight and beta diversity analysis of the microbiome of male SPF and ex-germ-free mice. (A) Body weight was measured on all SPF (*n* = 10) and ex-germ-free 1W (*n* = 14) and 3W (*n* = 11) mice during high-fat diet feeding and the average body weight is shown for each time point. Data points from mice that started losing weight because of diabetes were excluded. Error bars represent SD. (B, C) Principal coordinates analysis plots of nanopore-based sequencing of the near full 16S rRNA gene amplicon based on (B) Bray–Curtis (weighted) and (C) Jaccard (unweighted) distance matrices. The plots illustrate feces samples from SPF (*n* = 9, black) and 1W (*n* = 12, pink) and 3W (*n* = 10, purple) ex-germ-free mice at 10 week-of-age before start of high-fat diet. Figure S2: enrichment analysis pathways on genes differentially expressed in the liver. (A) Top 20 GO enriched pathways from Metascape. (B) Quality control of differential expressed genes showing enrichment in liver specific genes based on PaGenBase output from Metascape. (C) Enrichment mapped to human genes in Metascape showing enriched human diseases. Figure S3: liver cytokines in high-fat diet-induced male mice. (A–J) Liver cytokine and chemokine levels for nondiabetic SPF (*n* = 8), 1W (*n* = 9), and 3W (*n* = 9) mice were measured with mesoscale multiplex technology, and the analytes above detection range are shown as indicated. The concentrations were normalized to total protein measurements in the same samples. Mean and SEM are shown in all plots. ^∗^*p* value below 0.05. Table S1: differentially expressed genes in the liver. Output table from DESeq2 with differentially expressed genes in liver tissue from 3W (*n* = 5) male mice compared to SPF (*n* = 5) male mice, with an adjusted *p* value <0.05 and a fold-change <−1.5 or >1.5.

## References

[1] Bäckhed F., Ding H., Wang T. (2004). The gut microbiota as an environmental factor that regulates fat storage. *Proceedings of the National Academy of Sciences of the USA*.

[2] Moretti C. H., Schiffer T. A., Li X., Weitzberg E., Carlström M., Lundberg J. O. (2021). Germ-free mice are not protected against diet-induced obesity and metabolic dysfunction. *Acta Physiologica*.

[3] Fleissner C. K., Huebel N., Abd El-Bary M. M., Loh G., Klaus S., Blaut M. (2010). Absence of intestinal microbiota does not protect mice from diet-induced obesity. *British Journal of Nutrition*.

[4] Kübeck R., Bonet-Ripoll C., Hoffmann C. (2016). Dietary fat and gut microbiota interactions determine diet-induced obesity in mice. *Molecular Metabolism*.

[5] Logan I. E., Bobe G., Miranda C. L. (2020). Germ-free Swiss Webster mice on a high-fat diet develop obesity, hyperglycemia, and dyslipidemia. *Microorganisms*.

[6] Ley R. E., Backhed F., Turnbaugh P., Lozupone C. A., Knight R. D., Gordon J. I. (2005). Obesity alters gut microbial ecology. *Proceedings of the National Academy of Sciences*.

[7] Ley R. E., Turnbaugh P. J., Klein S., Gordon J. I. (2006). Microbial ecology: human gut microbes associated with obesity. *Nature*.

[8] Turnbaugh P. J., Hamady M., Yatsunenko T. (2009). A core gut microbiome in obese and lean twins. *Nature*.

[9] Tagliabue A., Elli M. (2013). The role of gut microbiota in human obesity: recent findings and future perspectives. *Nutrition, Metabolism, and Cardiovascular Diseases*.

[10] Di Ciaula A., Bonfrate L., Khalil M., Garruti G., Portincasa P. (2023). Contribution of the microbiome for better phenotyping of people living with obesity. *Reviews in Endocrine and Metabolic Disorders*.

[11] Ridaura V. K., Faith J. J., Rey F. E. (2013). Gut microbiota from twins discordant for obesity modulate metabolism in mice. *Science*.

[12] Turnbaugh P. J., Ley R. E., Mahowald M. A., Magrini V., Mardis E. R., Gordon J. I. (2006). An obesity-associated gut microbiome with increased capacity for energy harvest. *Nature*.

[13] Rabot S., Membrez M., Blancher F. (2016). High fat diet drives obesity regardless the composition of gut microbiota in mice. *Scientific Reports*.

[14] Lundberg R., Toft M. F., Metzdorff S. B. (2020). Human microbiota-transplanted C57BL/6 mice and offspring display reduced establishment of key bacteria and reduced immune stimulation compared to mouse microbiota-transplantation. *Scientific Reports*.

[15] Jian C., Carpén N., Helve O., de Vos W. M., Korpela K., Salonen A. (2021). Early-life gut microbiota and its connection to metabolic health in children: perspective on ecological drivers and need for quantitative approach. *EBioMedicine*.

[16] Cox L. M., Yamanishi S., Sohn J. (2014). Altering the intestinal microbiota during a critical developmental window has lasting metabolic consequences. *Cell*.

[17] Klancic T., Laforest-Lapointe I., Choo A. (2020). Prebiotic oligofructose prevents antibiotic-induced obesity risk and improves metabolic and gut microbiota profiles in rat dams and offspring. *Molecular Nutrition and Food Research*.

[18] Shao X., Ding X., Wang B. (2017). Antibiotic exposure in early life increases risk of childhood obesity: a systematic review and meta-analysis. *Frontiers in Endocrinology*.

[19] Cho I., Yamanishi S., Cox L. (2012). Antibiotics in early life alter the murine colonic microbiome and adiposity. *Nature*.

[20] Miao Z. H., Zhou W. X., Cheng R. Y. (2021). Dysbiosis of intestinal microbiota in early life aggravates high-fat diet induced dysmetabolism in adult mice. *BMC Microbiology*.

[21] Vandenplas Y., Carnielli V. P., Ksiazyk J. (2020). Factors affecting early-life intestinal microbiota development. *Nutrition*.

[22] Nobel Y. R., Cox L. M., Kirigin F. F. (2015). Metabolic and metagenomic outcomes from early-life pulsed antibiotic treatment. *Nature Communications*.

[23] Morel F. B., Oosting A., Piloquet H., Oozeer R., Darmaun D., Michel C. (2013). Can antibiotic treatment in preweaning rats alter body composition in adulthood?. *Neonatology*.

[24] Rune I., Hansen C. H., Ellekilde M. (2013). Ampicillin-improved glucose tolerance in diet-induced obese C57BL/6NTac mice is age dependent. *Journal of Diabetes Research*.

[25] Mozeš Š., Šefcíková Z., Bujnáková D., Racek L. (2013). Effect of antibiotic treatment on intestinal microbial and enzymatic development in postnatally overfed obese rats. *Obesity*.

[26] Pyndt Jørgensen B., Hansen J. T., Krych L. (2014). A possible link between food and mood: dietary impact on gut microbiota and behavior in BALB/c mice. *PLoS One*.

[27] Arildsen A. W., Zachariassen L. F., Krych L., Hansen A. K., Hansen C. H. F. (2021). Delayed gut colonization shapes future allergic responses in a murine model of atopic dermatitis. *Frontiers in Immunology*.

[28] https://www.ncbi.nlm.nih.gov/geo/query/acc.cgi?acc=GSE249397.

[29] Hansen C. H. F., Larsen C. S., Zachariassen L. F. (2022). Gluten-free diet reduces autoimmune diabetes mellitus in mice across multiple generations in a microbiota-independent manner. *Journal of Autoimmunity*.

[30] Ellekilde M., Krych L., Hansen C. H. (2014). Characterization of the gut microbiota in leptin deficient obese mice - correlation to inflammatory and diabetic parameters. *Research in Veterinary Science*.

[31] Bugianesi E., McCullough A. J., Marchesini G. (2005). Insulin resistance: a metabolic pathway to chronic liver disease. *Hepatology*.

[32] Bäckhed F., Roswall J., Peng Y. (2015). Dynamics and stabilization of the human gut microbiome during the first year of life. *Cell Host Microbe*.

[33] Sender R., Fuchs S., Milo R. (2016). Revised estimates for the number of human and bacteria cells in the body. *PLoS Biology*.

[34] Larsen N., Vogensen F. K., van den Berg F. W. (2010). Gut microbiota in human adults with type 2 diabetes differs from non-diabetic adults. *PLoS One*.

[35] Zhang M., Differding M. K., Benjamin-Neelon S. E., Østbye T., Hoyo C., Mueller N. T. (2019). Association of prenatal antibiotics with measures of infant adiposity and the gut microbiome. *Annals of Clinical Microbiology and Antimicrobials*.

[36] Korpela K., Zijlmans M. A., Kuitunen M. (2017). Childhood BMI in relation to microbiota in infancy and lifetime antibiotic use. *Microbiome*.

[37] Bäckhed F., Manchester J. K., Semenkovich C. F., Gordon J. I. (2007). Mechanisms underlying the resistance to diet-induced obesity in germ-free mice. *Proceedings of the National Academy of Sciences of the U S A*.

[38] Unger A. L., Eckstrom K., Jetton T. L., Kraft J. (2020). Facility-dependent metabolic phenotype and gut bacterial composition in CD-1 mice from a single vendor: a brief report. *PLoS One*.

[39] Müller V. M., Zietek T., Rohm F. (2016). Gut barrier impairment by high-fat diet in mice depends on housing conditions. *Molecular Nutrition and Food Research*.

[40] Montgomery M. K., Hallahan N. L., Brown S. H. (2013). Mouse strain-dependent variation in obesity and glucose homeostasis in response to high-fat feeding. *Diabetologia*.

[41] Lemke L. B., Rogers A. B., Nambiar P. R., Fox J. G. (2008). Obesity and non-insulin-dependent diabetes mellitus in Swiss-Webster mice associated with late-onset hepatocellular carcinoma. *Journal of Endocrinology*.

[42] Cordoba-Chacon J., Gahete M. D., Pozo-Salas A. I. (2012). Peripubertal-onset but not adult-onset obesity increases IGF-I and drives development of lean mass, which may lessen the metabolic impairment in adult obesity. *American Journal of Physiology-Endocrinology and Metabolism*.

[43] Hansen C. H., Nielsen D. S., Kverka M. (2012). Patterns of early gut colonization shape future immune responses of the host. *PLoS One*.

[44] Olszak T., An D., Zeissig S. (2012). Microbial exposure during early life has persistent effects on natural killer T cell function. *Science*.

[45] Ivanov I. I., Atarashi K., Manel N. (2009). Induction of intestinal Th17 cells by segmented filamentous bacteria. *Cell*.

[46] Oemcke L. A., Anderson R. C., Altermann E., Roy N. C., McNabb W. C. (2021). The role of segmented filamentous bacteria in immune barrier maturation of the small intestine at weaning. *Frontiers in Nutrition*.

[47] Delezie J., Dumont S., Dardente H. (2012). The nuclear receptor REV-ERB*α* is required for the daily balance of carbohydrate and lipid metabolism. *The FASEB Journal*.

[48] Marsal-Beltran A., Rodríguez-Castellano A., Astiarraga B. (2023). Protective effects of the succinate/SUCNR1 axis on damaged hepatocytes in NAFLD. *Metabolism*.

[49] Oberkofler H., Pfeifenberger A., Soyal S. (2010). Aberrant hepatic TRIB3 gene expression in insulin-resistant obese humans. *Diabetologia*.

[50] Patel P. R., Ramakrishnan S. K., Kaw M. K. (2012). Increased metabolic rate and insulin sensitivity in male mice lacking the carcino-embryonic antigen-related cell adhesion molecule 2. *Diabetologia*.

[51] Kaddai V., Jager J., Gonzalez T. (2009). Involvement of TNF-*α* in abnormal adipocyte and muscle sortilin expression in obese mice and humans. *Diabetologia*.

[52] Perry R. J., Samuel V. T., Petersen K. F., Shulman G. I. (2014). The role of hepatic lipids in hepatic insulin resistance and type 2 diabetes. *Nature*.

