# Supplementary material for: Delayed Gut Colonization Changes Future Insulin Resistance and Hepatic Gene Expression but Not Adiposity in Obese Mice
Source: J Obes. 2024 Sep 25;2024:5846674. doi: 10.1155/2024/5846674 (PMC11446614; doi:10.1155/2024/5846674)
Supplement: Supplementary Materials — Figure S1: body weight and beta diversity analysis of the microbiome of male SPF and ex-germ-free mice. (A) Body weight was measured on all SPF (n = 10) and ex-germ-free 1W (n = 14) and 3W (n = 11) mice during high-fat diet feeding and the average body weight is shown for each time point. Data points from mice that started losing weight because of diabetes were excluded. Error bars represent SD. (B, C) Principal coordinates analysis plots of nanopore-based sequencing of the near full 16S rRNA gene amplicon based on (B) Bray–Curtis (weighted) and (C) Jaccard (unweighted) distance matrices. The plots illustrate feces samples from SPF (n = 9, black) and 1W (n = 12, pink) and 3W (n = 10, purple) ex-germ-free mice at 10 week-of-age before start of high-fat diet. Figure S2: enrichment analysis pathways on genes differentially expressed in the liver. (A) Top 20 GO enriched pathways from Metascape. (B) Quality control of differential expressed genes showing enrichment in liver specific genes based on PaGenBase output from Metascape. (C) Enrichment mapped to human genes in Metascape showing enriched human diseases. Figure S3: liver cytokines in high-fat diet-induced male mice. (A–J) Liver cytokine and chemokine levels for nondiabetic SPF (n = 8), 1W (n = 9), and 3W (n = 9) mice were measured with mesoscale multiplex technology, and the analytes above detection range are shown as indicated. The concentrations were normalized to total protein measurements in the same samples. Mean and SEM are shown in all plots. ∗p value below 0.05. Table S1: differentially expressed genes in the liver. Output table from DESeq2 with differentially expressed genes in liver tissue from 3W (n = 5) male mice compared to SPF (n = 5) male mice, with an adjusted p value <0.05 and a fold-change <−1.5 or >1.5. [file 5846674.f1.zip › SupplementaryTable1 (1).pdf]

Supplementary table 1. Differentially Expressed Genes

Output table from DESeq2 with differentially expressed genesin liver tissue with an adjusted pvalue <0.05 and a foldchange <-1.5|>1.5

	Gene	baseMean	log2FoldChange	lfcSE	stat	pvalue	padj
1	Per3	323,07	3,39	0,18	18,83	4,31E-79	7,09E-75
2	Sort1	359,26	-2,68	0,15	-18,41	1,00E-75	8,25E-72
3	Dbp	329,66	6,04	0,33	18,25	1,95E-74	1,07E-70
4	Tef	1192,50	2,01	0,11	18,03	1,19E-72	4,88E-69
5	Mup11	4056,28	7,38	0,44	16,67	2,22E-62	7,30E-59
6	Nr1d1	957,85	2,08	0,16	13,11	3,03E-39	8,32E-36
7	Ppard	418,96	-2,07	0,16	-12,55	3,84E-36	9,03E-33
8	Nr1d2	1040,12	1,36	0,11	12,10	1,03E-33	2,12E-30
9	Nat8f5	100,44	-3,86	0,36	-10,74	6,32E-27	1,16E-23
10	Cyb561	81,75	-3,15	0,30	-10,50	8,46E-26	1,39E-22
11	Osbpl3	153,60	3,18	0,30	10,42	1,94E-25	2,90E-22
12	Zfp949	202,11	1,44	0,14	10,16	3,06E-24	4,20E-21
13	Dhx40	506,45	1,06	0,11	9,96	2,27E-23	2,87E-20
14	Gstm3	1838,66	-3,61	0,37	-9,84	7,70E-23	9,05E-20
15	B4galnt1	725,00	-1,10	0,11	-9,69	3,37E-22	3,70E-19
16	Gm8116	31,52	4,55	0,47	9,61	7,25E-22	7,45E-19
17	C1rl	1834,60	0,59	0,07	9,11	8,19E-20	7,93E-17
18	Gm43305	87,51	-2,51	0,28	-8,97	2,96E-19	2,70E-16
19	Dcaf7	851,89	-0,75	0,08	-8,89	6,34E-19	5,49E-16
20	Blm	135,98	1,86	0,21	8,79	1,44E-18	1,18E-15
21	Arntl	394,63	-1,14	0,13	-8,78	1,65E-18	1,29E-15
22	Ldlrad3	102,00	1,57	0,18	8,70	3,18E-18	2,38E-15
23	Mtss1	2218,33	0,85	0,10	8,63	6,41E-18	4,58E-15
24	Cdkn1a	209,27	-2,14	0,25	-8,57	1,03E-17	7,07E-15
25	Mup-ps20	55,98	8,22	0,96	8,56	1,18E-17	7,75E-15
26	Npas2	133,39	-2,55	0,30	-8,44	3,24E-17	2,05E-14
27	Gm11844	30,62	2,84	0,34	8,40	4,63E-17	2,82E-14
28	Mup15	5163,00	6,24	0,76	8,21	2,14E-16	1,26E-13
29	Nuggc	83,34	1,85	0,23	8,20	2,36E-16	1,34E-13
30	Thrsp	4330,70	1,85	0,23	8,14	3,93E-16	2,15E-13
31	Dtx4	465,78	-0,77	0,10	-8,02	1,02E-15	5,40E-13
32	Pnrc1	1597,83	-0,89	0,11	-7,91	2,64E-15	1,34E-12
33	Gm42944	44,28	3,47	0,44	7,90	2,69E-15	1,34E-12
34	Lonrf3	81,17	-1,58	0,20	-7,80	6,01E-15	2,91E-12
35	Mup7	10561,28	3,90	0,50	7,79	6,50E-15	3,06E-12
36	Cyp2c67	9628,81	0,84	0,11	7,70	1,35E-14	6,15E-12
37	Slco1a4	492,71	-1,87	0,24	-7,63	2,27E-14	1,01E-11
38	Mup20	28201,39	1,77	0,23	7,56	3,89E-14	1,69E-11
39	Per2	414,14	1,22	0,16	7,50	6,31E-14	2,66E-11
40	Abcg5	1000,15	0,82	0,11	7,49	6,66E-14	2,74E-11
41	Gm8189	40,58	1,87	0,25	7,47	7,84E-14	3,14E-11
42	Noct	524,67	1,73	0,23	7,45	9,09E-14	3,56E-11
43	Trim5	22,65	-7,26	0,98	-7,41	1,26E-13	4,81E-11
44	Mup-ps7	19,67	7,28	0,99	7,39	1,50E-13	5,60E-11
45	Crym	65,20	2,29	0,31	7,29	3,08E-13	1,13E-10
46	Gm26880	1408,07	-1,35	0,19	-7,29	3,19E-13	1,14E-10
47	Gm10382	126,47	-2,27	0,31	-7,26	3,80E-13	1,33E-10
48	Hsd3b5	5843,27	0,98	0,14	7,21	5,74E-13	1,95E-10
49	Pim1	398,77	1,12	0,16	7,21	5,79E-13	1,95E-10
50	Cry1	268,13	-1,26	0,18	-7,19	6,26E-13	2,06E-10
51	Gnat1	300,63	1,18	0,16	7,18	7,03E-13	2,27E-10

52	Cwc22	1128,38	-2,60	0,36	-7,17	7,75E-13	2,45E-10
53	Tff3	548,33	5,12	0,72	7,15	8,60E-13	2,67E-10
54	Gm7463	21,18	-4,56	0,64	-7,10	1,21E-12	3,69E-10
55	Slc13a4	40,97	2,60	0,37	7,05	1,76E-12	5,27E-10
56	Slc39a14	4044,65	0,72	0,10	7,01	2,32E-12	6,76E-10
57	Stard4	2735,00	0,61	0,09	7,01	2,34E-12	6,76E-10
58	Cyp2c55	63,04	-2,11	0,30	-6,97	3,11E-12	8,82E-10
59	4930404H11I	15,67	-6,73	0,97	-6,92	4,52E-12	1,26E-09
60	Celsr1	662,95	-0,83	0,12	-6,85	7,45E-12	2,04E-09
61	Rps26-ps1	42,13	-3,24	0,47	-6,84	7,77E-12	2,06E-09
62	Ugp2	3790,69	0,73	0,11	6,77	1,29E-11	3,38E-09
63	Zfp36l1	1804,57	-1,06	0,16	-6,70	2,08E-11	5,35E-09
64	Azin1	1405,59	0,68	0,10	6,69	2,30E-11	5,82E-09
65	Klhdc7a	985,83	0,71	0,11	6,66	2,74E-11	6,84E-09
66	Gm26588	87,94	1,15	0,17	6,66	2,78E-11	6,84E-09
67	Ank3	169,73	-1,46	0,22	-6,62	3,62E-11	8,76E-09
68	Fras1	293,27	-0,96	0,14	-6,62	3,69E-11	8,80E-09
69	Hsp90b1	13962,23	0,92	0,14	6,61	3,93E-11	9,16E-09
70	Mtnr1a	53,83	2,02	0,31	6,61	3,95E-11	9,16E-09
71	Tns2	1086,45	0,90	0,14	6,60	4,24E-11	9,69E-09
72	Hbb-bt	52,14	-2,84	0,43	-6,58	4,58E-11	1,03E-08
73	Cyp2c54	1331,08	1,36	0,21	6,58	4,77E-11	1,06E-08
74	Pdia3	9911,59	0,70	0,11	6,54	6,33E-11	1,39E-08
75	Gm1966	17,67	-5,76	0,89	-6,44	1,20E-10	2,59E-08
76	Mug-ps1	3135,89	0,83	0,13	6,39	1,67E-10	3,56E-08
77	Lgalsl	562,97	-1,00	0,16	-6,37	1,84E-10	3,89E-08
78	Tubb2a	1891,91	-1,15	0,18	-6,37	1,94E-10	4,04E-08
79	Gm11100	22,56	3,78	0,59	6,36	1,98E-10	4,06E-08
80	Mthfr	607,49	-1,31	0,21	-6,36	2,08E-10	4,23E-08
81	Spry4	182,69	1,35	0,21	6,32	2,58E-10	5,19E-08
82	Cib3	97,06	-2,25	0,36	-6,28	3,43E-10	6,80E-08
83	Upp2	2602,84	1,30	0,21	6,26	3,73E-10	7,31E-08
84	Them7	288,06	1,43	0,23	6,23	4,80E-10	9,21E-08
85	Pkdrej	117,81	1,59	0,26	6,23	4,81E-10	9,21E-08
86	Pdcd4	1297,61	0,59	0,09	6,22	5,01E-10	9,34E-08
87	Notch1	506,53	-0,85	0,14	-6,22	5,01E-10	9,34E-08
88	Gas5	402,00	0,66	0,11	6,22	5,05E-10	9,34E-08
89	Serpina12	2681,69	2,11	0,34	6,19	6,12E-10	1,12E-07
90	Klb	397,50	0,67	0,11	6,12	9,11E-10	1,65E-07
91	Dct	90,23	2,00	0,33	6,12	9,25E-10	1,65E-07
92	Desi2	604,31	-0,79	0,13	-6,11	9,99E-10	1,77E-07
93	A930036K24I	65,91	1,37	0,22	6,10	1,09E-09	1,91E-07
94	Inf2	910,37	1,34	0,22	6,06	1,36E-09	2,32E-07
95	Dnajc3	6718,31	0,79	0,13	6,05	1,43E-09	2,43E-07
96	Nbeal2	396,99	-0,92	0,15	-6,04	1,52E-09	2,54E-07
97	5830417I10R	22,50	3,94	0,65	6,03	1,61E-09	2,68E-07
98	Slc10a1	7043,97	0,74	0,12	6,03	1,63E-09	2,68E-07
99	Cited4	28,65	2,99	0,50	6,03	1,68E-09	2,74E-07
100	Jade1	814,35	0,70	0,12	6,02	1,73E-09	2,79E-07
101	Gm15408	66,59	1,41	0,23	6,02	1,76E-09	2,82E-07
102	Ciart	27,87	3,35	0,56	6,00	1,97E-09	3,11E-07
103	Abcg8	1533,90	0,78	0,13	5,97	2,43E-09	3,81E-07
104	Cyp7b1	13379,39	1,97	0,33	5,96	2,50E-09	3,88E-07
105	Slc2a2	4905,56	0,81	0,14	5,90	3,55E-09	5,46E-07
106	Mup-ps13	199,00	1,17	0,20	5,90	3,71E-09	5,61E-07
107	A930038B10	76,56	1,34	0,23	5,83	5,49E-09	8,21E-07
108	Stab2	1268,58	-0,66	0,11	-5,82	5,78E-09	8,57E-07

109	Armc10	189,74	-0,73	0,13	-5,77	8,07E-09	1,19E-06
110	Il15ra	583,26	0,71	0,12	5,72	1,06E-08	1,55E-06
111	Slco1b2	22122,10	0,77	0,13	5,71	1,11E-08	1,61E-06
112	Ndrg1	458,37	-1,23	0,22	-5,70	1,17E-08	1,67E-06
113	Gm3219	85,94	1,30	0,23	5,69	1,24E-08	1,77E-06
114	Etnk2	2044,38	0,90	0,16	5,67	1,46E-08	2,02E-06
115	Ccng2	309,23	-0,84	0,15	-5,66	1,51E-08	2,07E-06
116	Rpl19-ps11	132,32	1,82	0,32	5,66	1,53E-08	2,08E-06
117	Spcs3	1748,82	0,59	0,11	5,63	1,77E-08	2,39E-06
118	Trib3	81,63	1,45	0,26	5,59	2,24E-08	2,97E-06
119	Spon2	198,99	-1,45	0,26	-5,55	2,78E-08	3,56E-06
120	Hspa1b	501,29	-2,69	0,48	-5,55	2,79E-08	3,56E-06
121	Plcl2	172,29	1,43	0,26	5,54	3,07E-08	3,89E-06
122	Gm45301	26,09	2,78	0,51	5,50	3,74E-08	4,70E-06
123	Mycn	47,37	1,82	0,33	5,49	4,05E-08	5,01E-06
124	Sertad4	73,27	2,06	0,38	5,47	4,44E-08	5,46E-06
125	Akr1c14	6814,62	0,60	0,11	5,47	4,49E-08	5,47E-06
126	Ckap2	14,04	-2,65	0,49	-5,46	4,73E-08	5,68E-06
127	Itga6	78,22	1,03	0,19	5,46	4,73E-08	5,68E-06
128	Pxmp4	2253,53	-0,68	0,12	-5,45	5,08E-08	6,05E-06
129	Rabl3	268,58	0,74	0,14	5,44	5,19E-08	6,14E-06
130	Fancl	131,80	0,96	0,18	5,44	5,26E-08	6,18E-06
131	Cda	57,89	1,64	0,30	5,41	6,35E-08	7,40E-06
132	Cyp17a1	103,62	3,60	0,67	5,41	6,39E-08	7,40E-06
133	Gm31121	12,74	4,72	0,87	5,40	6,65E-08	7,60E-06
134	Lpl	866,89	-0,95	0,18	-5,40	6,84E-08	7,76E-06
135	Cyp2b10	17,08	-3,90	0,72	-5,39	7,13E-08	8,04E-06
136	Hspg2	865,43	-0,67	0,12	-5,38	7,59E-08	8,49E-06
137	Marco	48,67	-2,17	0,40	-5,37	7,72E-08	8,59E-06
138	Serpina5	114,82	1,77	0,33	5,37	8,00E-08	8,83E-06
139	Pebp1	4158,14	-0,94	0,18	-5,32	1,06E-07	1,15E-05
140	Slc37a4	2457,54	0,68	0,13	5,31	1,10E-07	1,18E-05
141	Cenpl	85,70	0,98	0,18	5,30	1,15E-07	1,22E-05
142	Rims2	9,88	-3,38	0,64	-5,28	1,26E-07	1,33E-05
143	Mup-ps6	8,89	4,54	0,86	5,25	1,51E-07	1,57E-05
144	Igfbp5	275,17	0,74	0,14	5,23	1,67E-07	1,71E-05
145	Smpd3	48,37	2,53	0,48	5,22	1,74E-07	1,77E-05
146	Stab1	1018,89	-0,63	0,12	-5,22	1,84E-07	1,85E-05
147	Mup-ps15	32,89	2,00	0,38	5,21	1,86E-07	1,87E-05
148	Mcm6	83,88	-0,99	0,19	-5,20	1,95E-07	1,95E-05
149	Igha	57,91	-2,06	0,40	-5,20	2,00E-07	1,98E-05
150	Gm6135	481,14	4,66	0,90	5,18	2,22E-07	2,18E-05
151	Asap2	372,83	-0,80	0,15	-5,17	2,37E-07	2,31E-05
152	Tes	39,47	1,51	0,29	5,16	2,44E-07	2,36E-05
153	Frmd4b	1068,50	0,98	0,19	5,16	2,46E-07	2,37E-05
154	Nucb2	146,27	1,52	0,29	5,16	2,53E-07	2,42E-05
155	Gm30551	49,83	1,06	0,21	5,14	2,73E-07	2,58E-05
156	Ptprb	1556,92	-0,63	0,12	-5,13	2,90E-07	2,72E-05
157	Ppat	368,16	0,84	0,16	5,12	2,98E-07	2,79E-05
158	Chka	2094,78	-1,02	0,20	-5,12	3,12E-07	2,90E-05
159	Gm26684	143,49	1,07	0,21	5,10	3,35E-07	3,10E-05
160	Slc15a5	29,07	1,70	0,33	5,10	3,41E-07	3,14E-05
161	Tmem184c	271,49	1,17	0,23	5,06	4,13E-07	3,76E-05
162	Brip1os	320,59	0,67	0,13	5,06	4,19E-07	3,76E-05
163	A530001N23	24,00	-1,76	0,35	-5,05	4,35E-07	3,89E-05
164	Dpy19l3	661,39	0,88	0,17	5,05	4,47E-07	3,97E-05
165	Cyp2u1	1099,84	0,84	0,17	5,04	4,57E-07	4,04E-05

166	Trim6	66,34	-1,24	0,25	-5,03	4,82E-07	4,24E-05
167	Gm12718	135,35	1,22	0,24	5,03	4,99E-07	4,37E-05
168	Pdk1	843,75	0,82	0,16	5,00	5,70E-07	4,91E-05
169	Bnc2	12,86	4,74	0,95	5,00	5,78E-07	4,95E-05
170	Zfp953	54,12	1,13	0,23	4,99	6,04E-07	5,15E-05
171	Mup-ps8	7,07	5,20	1,05	4,97	6,57E-07	5,57E-05
172	Gm31522	19,88	1,71	0,34	4,97	6,69E-07	5,65E-05
173	Rgs16	192,43	4,30	0,87	4,95	7,47E-07	6,27E-05
174	F8	343,42	-0,74	0,15	-4,95	7,58E-07	6,33E-05
175	Rgs7	44,71	4,07	0,82	4,94	7,80E-07	6,45E-05
176	Setd4	153,33	0,95	0,19	4,94	8,00E-07	6,58E-05
177	Dgka	159,77	-0,84	0,17	-4,93	8,06E-07	6,60E-05
178	Gm37729	113,96	1,08	0,22	4,92	8,87E-07	7,17E-05
179	Gm37317	8,34	4,87	0,99	4,91	8,89E-07	7,17E-05
180	Clpx	4474,73	-0,70	0,14	-4,89	9,99E-07	7,98E-05
181	Isyna1	1337,72	4,34	0,89	4,89	1,02E-06	8,09E-05
182	Saa2	128,71	-0,92	0,19	-4,89	1,03E-06	8,12E-05
183	Tmem140	136,26	-0,66	0,13	-4,87	1,10E-06	8,58E-05
184	Tinagl1	289,40	-0,69	0,14	-4,87	1,14E-06	8,88E-05
185	Calcr	10,21	2,70	0,56	4,86	1,20E-06	9,21E-05
186	Zbtb37	254,67	0,95	0,20	4,84	1,27E-06	9,76E-05
187	Moxd1	85,76	8,32	1,72	4,83	1,34E-06	0,00010246
188	Gpcpd1	1707,72	2,09	0,43	4,82	1,41E-06	0,00010704
189	Rtp3	557,45	-0,65	0,13	-4,81	1,47E-06	0,00011065
190	Rpl13-ps2	7,53	4,73	0,99	4,79	1,65E-06	0,00012289
191	Bmf	148,15	1,29	0,27	4,78	1,73E-06	0,00012764
192	Mat1a	27252,03	0,73	0,15	4,78	1,77E-06	0,00013008
193	Prc1	15,31	-1,87	0,39	-4,77	1,86E-06	0,00013624
194	Cdcp1	109,57	0,94	0,20	4,76	1,95E-06	0,00014166
195	Ccnd1	471,67	-1,68	0,35	-4,74	2,13E-06	0,00015455
196	Cyp2c37	315,13	0,97	0,20	4,74	2,17E-06	0,00015631
197	Dnajb1	449,32	-1,07	0,23	-4,73	2,22E-06	0,00015948
198	Camkk2	287,67	-0,64	0,13	-4,73	2,28E-06	0,00016335
199	Adra1a	55,50	-0,99	0,21	-4,72	2,37E-06	0,000169
200	Gm43597	79,45	-0,97	0,21	-4,71	2,43E-06	0,00017234
201	Gm26669	66,20	-1,73	0,37	-4,71	2,47E-06	0,00017346
202	Stap1	26,24	1,43	0,30	4,71	2,50E-06	0,0001747
203	Dnajb11	1945,38	0,67	0,14	4,71	2,51E-06	0,0001747
204	Gm4316	357,58	2,26	0,48	4,70	2,58E-06	0,0001789
205	Omd	34,82	1,97	0,42	4,68	2,83E-06	0,00019549
206	Plxnd1	757,59	-0,65	0,14	-4,68	2,88E-06	0,00019826
207	Iqcn	22,67	1,53	0,33	4,68	2,93E-06	0,00020059
208	Wfdc15b	14,20	2,97	0,64	4,67	3,00E-06	0,00020501
209	Kctd1	26,10	1,53	0,33	4,67	3,02E-06	0,00020528
210	Slc2a5	86,57	1,11	0,24	4,67	3,06E-06	0,00020613
211	Fam98a	765,06	0,74	0,16	4,67	3,07E-06	0,00020613
212	Arhgap26	606,98	0,62	0,13	4,66	3,09E-06	0,0002067
213	Serpina1e	29425,14	-4,93	1,06	-4,66	3,22E-06	0,00021467
214	Dsg1c	327,34	1,72	0,37	4,65	3,32E-06	0,00021909
215	Fam89a	137,63	-1,41	0,30	-4,65	3,33E-06	0,00021909
216	1700086O06	22,35	1,82	0,39	4,65	3,36E-06	0,0002205
217	Ncald	238,56	-0,72	0,15	-4,64	3,45E-06	0,00022495
218	Sigmar1	3363,59	-0,63	0,14	-4,64	3,48E-06	0,00022588
219	Scara5	308,05	1,93	0,42	4,64	3,49E-06	0,00022588
220	Rhbdf2	342,22	-0,69	0,15	-4,63	3,62E-06	0,0002336
221	Cyp2c50	3031,55	0,85	0,18	4,62	3,87E-06	0,00024673
222	Serpine2	16,89	1,77	0,38	4,61	3,93E-06	0,00024897

223	Vegfb	180,28	-0,83	0,18	-4,61	4,07E-06	0,00025603
224	Mep1b	10,28	3,09	0,67	4,61	4,08E-06	0,00025603
225	Sirt5	326,71	-0,93	0,20	-4,60	4,13E-06	0,00025827
226	Gm5524	271,57	0,63	0,14	4,60	4,21E-06	0,00026244
227	Cxcl14	38,52	3,55	0,77	4,60	4,30E-06	0,000267
228	9330159M07	20,49	-1,52	0,33	-4,60	4,32E-06	0,00026705
229	Tjp3	563,75	-0,65	0,14	-4,59	4,34E-06	0,0002676
230	Mad2l2	283,31	-0,60	0,13	-4,59	4,43E-06	0,00027206
231	Ero1lb	1809,49	0,71	0,16	4,58	4,59E-06	0,00028081
232	Nt5dc2	113,88	-0,74	0,16	-4,58	4,61E-06	0,00028081
233	Pamr1	35,24	-1,31	0,29	-4,58	4,63E-06	0,00028098
234	Tmem132e	58,89	-0,85	0,19	-4,58	4,67E-06	0,00028148
235	Tead1	95,54	-0,83	0,18	-4,58	4,70E-06	0,0002824
236	Mup1	253,79	1,29	0,28	4,58	4,75E-06	0,00028398
237	Hsf2bp	29,68	1,83	0,40	4,56	5,07E-06	0,00029917
238	Nat8f6	11,28	-2,49	0,55	-4,56	5,21E-06	0,00030594
239	Hspa1a	131,73	-2,57	0,56	-4,55	5,29E-06	0,00030972
240	4930452B06I	17,70	1,61	0,36	4,54	5,67E-06	0,00032962
241	Ift57	27,36	-1,62	0,36	-4,54	5,73E-06	0,00033202
242	Nat8	173,75	-0,83	0,18	-4,51	6,59E-06	0,00037879
243	Gm5619	44,83	7,89	1,75	4,51	6,61E-06	0,00037879
244	Gm14097	43,85	3,96	0,88	4,50	6,71E-06	0,00038206
245	Fam222a	132,19	-0,95	0,21	-4,49	7,00E-06	0,00039707
246	Gm43593	27,58	1,39	0,31	4,47	7,76E-06	0,00043604
247	Clint1	1115,96	-0,62	0,14	-4,46	8,10E-06	0,00045324
248	Gm12250	61,49	1,00	0,23	4,45	8,41E-06	0,00046751
249	Klf10	248,20	1,14	0,26	4,45	8,71E-06	0,00048259
250	Fkbp11	312,95	0,95	0,21	4,44	9,19E-06	0,00050467
251	Myh7b	8,10	-2,91	0,66	-4,42	9,71E-06	0,00052897
252	Gm19950	186,39	0,83	0,19	4,42	9,75E-06	0,00052966
253	Gm11266	35,03	1,46	0,33	4,42	1,00E-05	0,00054183
254	Derl3	220,44	2,33	0,53	4,40	1,08E-05	0,0005754
255	Ksr1	52,07	-0,87	0,20	-4,40	1,09E-05	0,00057552
256	Aox1	4362,42	-0,75	0,17	-4,40	1,09E-05	0,00057552
257	Pklr	2650,57	0,71	0,16	4,40	1,11E-05	0,00058211
258	Gm15611	48,21	-1,54	0,35	-4,39	1,13E-05	0,0005897
259	Trim30d	23,02	-1,64	0,37	-4,38	1,18E-05	0,00061404
260	Notch4	100,36	-0,72	0,16	-4,36	1,30E-05	0,00066804
261	Adgrv1	308,67	1,07	0,25	4,36	1,31E-05	0,00067326
262	Gm1818	8,21	2,81	0,65	4,35	1,38E-05	0,00070672
263	Zbtb21	270,26	-0,71	0,16	-4,35	1,39E-05	0,00071021
264	Ube2d2b	17,32	-1,56	0,36	-4,34	1,41E-05	0,00072007
265	Loxl4	22,75	-1,51	0,35	-4,34	1,44E-05	0,00073004
266	Amigo1	174,24	-0,74	0,17	-4,34	1,46E-05	0,0007344
267	Mtx3	390,03	0,62	0,14	4,33	1,48E-05	0,00074438
268	mt-Tw	26,73	3,59	0,83	4,33	1,48E-05	0,00074438
269	Ccnd2	213,34	-0,59	0,14	-4,33	1,52E-05	0,00075724
270	Serpina3a	20,94	2,19	0,51	4,32	1,57E-05	0,00077864
271	Top2a	42,26	-1,16	0,27	-4,32	1,58E-05	0,00078132
272	Mup3	106740,20	0,76	0,18	4,32	1,59E-05	0,00078468
273	Gm14403	298,96	1,17	0,27	4,31	1,61E-05	0,00078844
274	Paxx	330,29	-0,83	0,19	-4,31	1,65E-05	0,00080644
275	Nbas	825,05	0,62	0,14	4,30	1,67E-05	0,00081078
276	Papln	25,51	-1,59	0,37	-4,30	1,68E-05	0,00081249
277	Tedc2	320,63	0,64	0,15	4,30	1,71E-05	0,0008232
278	Kyat3	2148,31	-0,69	0,16	-4,29	1,80E-05	0,00086564
279	Atp6v1g2	20,09	-1,40	0,33	-4,28	1,84E-05	0,00088426

280	Mn1	319,88	0,65	0,15	4,28	1,90E-05	0,00090444
281	Efna3	54,01	-0,91	0,21	-4,28	1,90E-05	0,00090444
282	Gm9118	37,62	2,04	0,48	4,27	1,91E-05	0,00090719
283	Oit3	418,32	-0,64	0,15	-4,27	1,93E-05	0,00091026
284	Gm3226	10,31	-3,89	0,91	-4,26	2,06E-05	0,0009645
285	Gna14	83,29	0,96	0,22	4,25	2,11E-05	0,00098158
286	Rpl7a-ps3	5,69	4,87	1,15	4,25	2,17E-05	0,0010096
287	Crot	9796,57	-0,59	0,14	-4,24	2,19E-05	0,001014
288	Ceacam2	246,68	-5,42	1,28	-4,23	2,31E-05	0,0010668
289	Slc9a9	73,68	-0,77	0,18	-4,23	2,34E-05	0,00107354
290	Fam43a	111,72	-0,68	0,16	-4,23	2,34E-05	0,00107354
291	Mup21	10017,80	2,02	0,48	4,23	2,35E-05	0,00107354
292	Mmp15	1037,79	-0,59	0,14	-4,23	2,37E-05	0,00108018
293	N4bp2l1	793,28	0,66	0,16	4,22	2,44E-05	0,00111057
294	Gm15889	26,47	1,75	0,42	4,22	2,49E-05	0,00112565
295	E230016M11	54,96	0,80	0,19	4,22	2,49E-05	0,00112565
296	Ptges	10,71	2,21	0,52	4,21	2,52E-05	0,00113581
297	Ces2a	6814,28	-0,73	0,17	-4,21	2,55E-05	0,00114455
298	4930455J16R	6,22	4,41	1,05	4,21	2,59E-05	0,00116245
299	Tpx2	20,35	-1,87	0,45	-4,20	2,70E-05	0,00119983
300	Ica1	62,73	1,43	0,34	4,19	2,74E-05	0,00120065
301	H2-T24	130,46	-1,26	0,30	-4,19	2,74E-05	0,00120065
302	Atad2	96,14	-0,67	0,16	-4,19	2,75E-05	0,00120065
303	Hspa2	32,94	-1,20	0,29	-4,19	2,76E-05	0,00120065
304	Pdgfc	130,50	-0,59	0,14	-4,19	2,76E-05	0,00120065
305	Lmf1	794,61	0,62	0,15	4,19	2,77E-05	0,00120334
306	Gm21320	416,85	1,58	0,38	4,18	2,92E-05	0,00125334
307	Sult2a8	17144,87	0,72	0,17	4,17	3,04E-05	0,00130188
308	Nt5e	343,08	-0,73	0,17	-4,16	3,15E-05	0,00134636
309	Glt1d1	207,70	0,86	0,21	4,16	3,17E-05	0,00134962
310	Gm44243	101,25	-1,29	0,31	-4,16	3,19E-05	0,001358
311	Ppp1r3c	1696,88	-0,96	0,23	-4,15	3,27E-05	0,00138575
312	Vnn3	222,03	-0,86	0,21	-4,15	3,35E-05	0,00141302
313	Ap3m1	1610,91	0,62	0,15	4,14	3,44E-05	0,00144839
314	Zbtb40	192,74	-0,67	0,16	-4,14	3,49E-05	0,00146318
315	Cd209g	5,21	-5,12	1,24	-4,13	3,57E-05	0,00149013
316	Mup-ps10	16,87	-4,58	1,11	-4,13	3,62E-05	0,00149456
317	Nat8f4	145,58	0,94	0,23	4,13	3,67E-05	0,00151492
318	Bag3	515,14	-0,70	0,17	-4,12	3,73E-05	0,0015322
319	Tmie	176,87	0,93	0,23	4,11	3,95E-05	0,00161139
320	Mast3	644,49	0,59	0,14	4,11	4,02E-05	0,00163911
321	Gm44096	22,42	1,18	0,29	4,09	4,24E-05	0,00171017
322	BC048644	79,33	1,07	0,26	4,09	4,34E-05	0,00174224
323	Gm16249	3,63	-5,20	1,27	-4,08	4,49E-05	0,00178256
324	Ccdc117	486,06	-0,66	0,16	-4,08	4,60E-05	0,0018189
325	2310001H17I	296,01	0,67	0,17	4,07	4,61E-05	0,00181962
326	Asns	69,75	1,63	0,40	4,07	4,70E-05	0,00184061
327	Gadd45a	37,93	1,61	0,39	4,07	4,71E-05	0,00184061
328	Gm44851	12,97	1,82	0,45	4,06	4,96E-05	0,00193038
329	Neurl1b	20,39	-1,37	0,34	-4,05	5,07E-05	0,00196345
330	Ube2c	24,18	-1,66	0,41	-4,05	5,08E-05	0,00196345
331	Ms4a8a	12,34	-2,47	0,61	-4,04	5,26E-05	0,00201594
332	Capn8	420,39	2,43	0,60	4,04	5,33E-05	0,00203691
333	Eln	131,73	-0,95	0,23	-4,04	5,34E-05	0,00203691
334	Necab1	705,07	0,66	0,16	4,04	5,35E-05	0,00203695
335	Spp1	812,70	1,81	0,45	4,04	5,36E-05	0,00203818
336	Pde4a	95,36	-0,92	0,23	-4,03	5,51E-05	0,00208295

337	Soga1	67,58	-0,91	0,23	-4,03	5,68E-05	0,00213833
338	Miox	8,56	3,43	0,85	4,02	5,72E-05	0,00213975
339	Spon1	161,35	3,75	0,93	4,02	5,73E-05	0,00213975
340	Sema3f	139,11	-0,67	0,17	-4,02	5,74E-05	0,00213975
341	Slc16a5	62,32	-1,44	0,36	-4,02	5,76E-05	0,00213975
342	Pdia4	4875,26	0,60	0,15	4,02	5,94E-05	0,00220212
343	Ephx1	6636,47	-0,71	0,18	-4,00	6,28E-05	0,00231191
344	Gm15998	123,12	-1,03	0,26	-4,00	6,38E-05	0,0023406
345	Chst12	29,01	-1,10	0,27	-4,00	6,39E-05	0,0023406
346	H2-K1	3369,26	0,92	0,23	4,00	6,40E-05	0,0023406
347	Lamb2	119,25	-0,79	0,20	-3,99	6,68E-05	0,00243149
348	Bach2	232,87	0,83	0,21	3,99	6,70E-05	0,00243259
349	1810055G02	327,71	1,07	0,27	3,98	6,84E-05	0,00246377
350	Sult1b1	381,18	0,78	0,20	3,98	6,93E-05	0,00248799
351	Gm15318	123,31	0,91	0,23	3,98	7,03E-05	0,00251777
352	Acot2	33,84	-1,70	0,43	-3,97	7,06E-05	0,00251777
353	A730013G03	14,17	1,96	0,49	3,97	7,30E-05	0,00257639
354	Gm30262	350,34	0,62	0,16	3,96	7,44E-05	0,00260983
355	Rhoc	199,32	-1,23	0,31	-3,96	7,50E-05	0,00262622
356	St5	819,75	-0,68	0,17	-3,95	7,79E-05	0,00271708
357	Cyp2j6	1179,17	0,60	0,15	3,95	7,93E-05	0,00275937
358	Cry2	521,71	0,63	0,16	3,95	7,96E-05	0,00276028
359	Mtss1l	67,15	-0,81	0,20	-3,95	7,97E-05	0,00276028
360	Gm45609	13,96	1,98	0,50	3,93	8,33E-05	0,00286133
361	Fam107a	5,62	3,83	0,97	3,93	8,42E-05	0,00288564
362	Ces1d	6902,81	-1,31	0,33	-3,91	9,07E-05	0,00308994
363	Sucnr1	300,17	1,48	0,38	3,91	9,30E-05	0,00315665
364	Oat	6918,17	0,72	0,18	3,91	9,34E-05	0,00316303
365	Tiam2	63,65	0,95	0,24	3,90	9,74E-05	0,00327618
366	Gm8822	65,02	0,98	0,25	3,90	9,77E-05	0,00328035
367	Dkk3	34,04	-1,10	0,28	-3,88	0,00010299	0,00345139
368	Prom2	9,08	-3,08	0,79	-3,88	0,00010331	0,003455
369	Palld	1470,00	-0,60	0,16	-3,88	0,00010399	0,00347085
370	Dusp8	53,76	-1,72	0,44	-3,88	0,00010598	0,00352295
371	Cyp2d36-ps	473,22	0,97	0,25	3,87	0,00011033	0,00365998
372	Myo15b	15,61	1,68	0,44	3,86	0,0001117	0,00369813
373	Cd276	91,95	-0,90	0,23	-3,86	0,00011242	0,00371448
374	Sipa1l2	128,26	0,62	0,16	3,86	0,00011368	0,00374856
375	Adamts10	132,65	-0,72	0,19	-3,86	0,00011452	0,00376103
376	Zfp105	12,10	1,71	0,44	3,86	0,0001151	0,0037699
377	Rnf225	60,11	0,94	0,25	3,85	0,00011655	0,00379622
378	She	104,77	-0,66	0,17	-3,85	0,0001197	0,00386942
379	Per1	283,56	2,26	0,59	3,84	0,00012106	0,00390562
380	Sdf2l1	922,21	0,89	0,23	3,84	0,0001231	0,00396373
381	Sec11c	508,49	0,59	0,15	3,84	0,00012335	0,00396399
382	Gramd4	155,42	-0,72	0,19	-3,83	0,00012716	0,00407076
383	Fhod1	150,94	-0,60	0,16	-3,83	0,00012973	0,00414493
384	Ccnb1	15,03	-2,13	0,56	-3,83	0,00013011	0,0041488
385	Axin2	172,67	-0,60	0,16	-3,82	0,00013445	0,00426258
386	Gata2	17,24	-1,37	0,36	-3,81	0,00013727	0,00434357
387	Cacul1	1101,48	0,62	0,16	3,81	0,00013796	0,0043515
388	Igkc	72,77	-0,91	0,24	-3,81	0,00013805	0,0043515
389	Shank3	145,36	-0,69	0,18	-3,80	0,00014457	0,00452466
390	Cenpe	12,48	-1,88	0,49	-3,80	0,00014464	0,00452466
391	Nrg1	13,37	2,11	0,55	3,80	0,00014581	0,00455258
392	Gm31583	1695,34	1,28	0,34	3,79	0,00014897	0,00464226
393	Gm45769	46,22	1,28	0,34	3,79	0,00015202	0,00471963

394	Clic3	69,30	-2,09	0,55	-3,78	0,00015659	0,00484299
395	Egfr	15280,56	0,80	0,21	3,77	0,00016315	0,00502696
396	Klf15	2382,65	0,60	0,16	3,77	0,00016613	0,00510937
397	Map4k4	202,10	-0,61	0,16	-3,76	0,00017174	0,00527215
398	Bcl6b	39,16	-0,85	0,23	-3,76	0,00017312	0,00529759
399	Gfod1	86,34	-0,89	0,24	-3,75	0,00017467	0,00532241
400	Grk3	104,23	-1,19	0,32	-3,75	0,00017644	0,00536614
401	Cd14	79,62	1,16	0,31	3,75	0,00017947	0,00544833
402	2810021J22R	81,72	0,72	0,19	3,75	0,00018039	0,00545339
403	Gm23726	55,03	2,25	0,60	3,74	0,00018056	0,00545339
404	F730311O21	15,76	-1,67	0,45	-3,74	0,0001826	0,00550286
405	Scnn1a	454,72	-0,66	0,18	-3,74	0,00018548	0,00556899
406	Peli3	37,84	-1,10	0,29	-3,73	0,00018932	0,00566381
407	Gm8834	10,25	-1,82	0,49	-3,73	0,0001909	0,00569021
408	Srgap3	42,30	1,06	0,28	3,73	0,00019168	0,00570314
409	Gpc1	114,15	-0,89	0,24	-3,73	0,00019257	0,00571952
410	Fmn2	110,66	1,26	0,34	3,72	0,00019629	0,00581138
411	Gm8430	24,48	1,32	0,36	3,72	0,00019637	0,00581138
412	Chst8	25,38	7,65	2,06	3,72	0,00019959	0,00589599
413	Pik3c2g	605,55	0,92	0,25	3,72	0,00019999	0,00589724
414	A930033H14	44,33	2,72	0,73	3,71	0,00020637	0,0060419
415	Gm3776	15,89	-2,85	0,77	-3,71	0,00020988	0,00613373
416	4933417D19I	10,08	2,56	0,69	3,70	0,0002119	0,0061535
417	Nlrc3	25,26	-1,17	0,31	-3,70	0,00021205	0,0061535
418	Adamts5	59,95	-0,86	0,23	-3,70	0,00021296	0,00616904
419	Nos3	52,80	-0,84	0,23	-3,70	0,00021556	0,0062054
420	Tnfrsf14	106,36	-0,65	0,18	-3,70	0,00021908	0,00627993
421	Gm39318	10,75	2,12	0,57	3,69	0,0002237	0,00640122
422	Ppp1r1b	50,39	-1,24	0,34	-3,69	0,00022585	0,00645164
423	Rab3il1	69,98	-0,73	0,20	-3,69	0,00022813	0,00649421
424	Gm4070	15,29	1,40	0,38	3,69	0,00022862	0,00649679
425	Robo4	146,88	-0,69	0,19	-3,68	0,00022959	0,00651316
426	Tmc5	6,49	2,77	0,75	3,68	0,0002313	0,00655051
427	Zmym1	46,30	-0,76	0,21	-3,67	0,00023919	0,00676229
428	Mup-ps12	76,41	1,32	0,36	3,67	0,00023988	0,00677027
429	Gm35339	71,38	-0,70	0,19	-3,67	0,00024133	0,0067995
430	Gm14287	14,65	-2,21	0,60	-3,66	0,00024916	0,00698404
431	St3gal5	832,76	0,62	0,17	3,66	0,00025153	0,00703863
432	Gm34777	26,28	1,18	0,32	3,66	0,00025317	0,00706759
433	D030055H07	27,49	-0,95	0,26	-3,66	0,00025343	0,00706759
434	Hs3st3a1	79,91	-1,12	0,31	-3,65	0,00026366	0,00730356
435	Phospho1	17,58	-1,40	0,38	-3,65	0,00026656	0,00735908
436	Ckap2l	10,54	-2,09	0,57	-3,65	0,00026735	0,00736848
437	Mup8	88,88	2,89	0,79	3,64	0,00027282	0,00749413
438	Gm45221	21,31	1,94	0,53	3,64	0,00027355	0,00750152
439	Gm13727	3,64	-3,95	1,09	-3,63	0,00028677	0,00781083
440	mt-Tn	53,09	1,56	0,43	3,62	0,00029013	0,00787194
441	Gm27252	23,98	1,11	0,31	3,62	0,00029774	0,00804422
442	Wdr5b	43,46	0,76	0,21	3,62	0,00029922	0,00807121
443	Pde4d	85,72	-1,14	0,32	-3,61	0,00030068	0,00809719
444	F11	2783,63	0,60	0,17	3,61	0,00030333	0,00815524
445	Herpud1	4655,45	0,74	0,20	3,61	0,00030586	0,00818313
446	mt-Cytb	87641,94	0,65	0,18	3,61	0,00030695	0,00819883
447	Ces1g	2613,69	-0,72	0,20	-3,61	0,00031145	0,00827884
448	Aspm	14,36	-1,66	0,46	-3,60	0,00031268	0,00829815
449	Npl	43,54	-0,85	0,24	-3,60	0,00031485	0,00834219
450	Pmvk	621,16	-0,78	0,22	-3,60	0,00032036	0,00843403

451	H2-Ab1	69,75	-0,73	0,20	-3,59	0,00033629	0,00876904
452	Capn3	115,87	1,14	0,32	3,58	0,00033745	0,00878546
453	Pwwp2b	54,43	-0,84	0,24	-3,58	0,00033857	0,00878683
454	A230103J11R	7,94	-2,16	0,60	-3,58	0,00034409	0,00887403
455	Nptx1	12,81	1,52	0,42	3,58	0,00034674	0,00890052
456	Troap	4,56	-2,92	0,82	-3,58	0,00034906	0,00894606
457	Hopx	207,15	0,77	0,21	3,57	0,00035785	0,00913209
458	Gm38832	27,99	1,57	0,44	3,57	0,00035798	0,00913209
459	Nfkb2	143,89	-0,68	0,19	-3,57	0,00036067	0,0091866
460	Gm17040	3,01	5,16	1,45	3,56	0,00036652	0,00930228
461	Cyp21a1	21,00	1,64	0,46	3,56	0,00036656	0,00930228
462	Tcf7	76,49	-0,60	0,17	-3,56	0,0003685	0,00932803
463	Brca2	100,21	0,64	0,18	3,56	0,00037055	0,0093656
464	Sptssa	1326,09	1,74	0,49	3,56	0,00037124	0,00936877
465	Hsbp1l1	85,97	0,81	0,23	3,56	0,00037271	0,00938445
466	Fads3	320,37	1,39	0,39	3,55	0,00038012	0,00953437
467	Chad	121,87	-0,98	0,28	-3,55	0,00038206	0,0095683
468	Tppp	179,74	-0,64	0,18	-3,55	0,00038519	0,00963214
469	Ccna2	21,92	-1,35	0,38	-3,55	0,00038606	0,00963929
470	Lgals1	62,39	-1,17	0,33	-3,55	0,00038837	0,00968221
471	Tuba1a	117,86	-1,72	0,49	-3,54	0,00039384	0,0098036
472	Tceal8	305,97	0,76	0,22	3,54	0,00039455	0,00980439
473	Retsat	2987,73	-0,71	0,20	-3,54	0,00039595	0,00980439
474	Tspan18	49,69	-0,73	0,21	-3,54	0,00039643	0,00980439
475	Dysf	116,30	-0,71	0,20	-3,54	0,00039685	0,00980439
476	E2f7	13,80	1,57	0,44	3,54	0,00040024	0,00987334
477	Piezo1	141,44	-0,67	0,19	-3,54	0,0004076	0,00998206
478	2310057J18R	40,69	5,96	1,69	3,53	0,00041162	0,01004858
479	Gm42743	9,35	-2,70	0,77	-3,53	0,00041779	0,01016916
480	Fscn1	87,67	-0,70	0,20	-3,52	0,00042352	0,01027827
481	Hmcn1	78,99	-0,67	0,19	-3,52	0,00042562	0,01031382
482	AA986860	21,20	1,17	0,33	3,52	0,00042685	0,01032849
483	F2r	389,21	0,80	0,23	3,52	0,00043695	0,01055744
484	Ankrd31	5,16	2,84	0,81	3,51	0,00044039	0,01060932
485	Ulk1	1859,10	0,60	0,17	3,50	0,00046356	0,01112065
486	Gm13178	7,92	2,05	0,59	3,50	0,00047062	0,01127149
487	Plpp7	15,71	1,39	0,40	3,49	0,00047944	0,01144955
488	Ttbk1	28,49	1,59	0,46	3,49	0,00048377	0,01151935
489	Hecw2	74,23	-0,61	0,18	-3,48	0,00049466	0,01174468
490	Hspd1-ps4	18,53	6,61	1,90	3,48	0,00049658	0,01177327
491	Gm17662	33,52	0,90	0,26	3,48	0,00050936	0,01192188
492	Tbx3os1	90,34	0,79	0,23	3,47	0,00052306	0,0121732
493	Adamts7	280,51	0,74	0,21	3,47	0,0005256	0,01221499
494	Vmn2r20	36,98	1,81	0,52	3,47	0,00052799	0,01225316
495	P4ha1	252,68	-0,69	0,20	-3,46	0,00053377	0,01235256
496	Gm11419	24,31	1,27	0,37	3,46	0,00053558	0,01237692
497	Trim12c	347,16	1,34	0,39	3,45	0,00055148	0,01265561
498	Gm15502	29,20	1,07	0,31	3,45	0,00055497	0,01270034
499	Mug1	121526,91	0,64	0,19	3,45	0,00056685	0,01295416
500	Mcm10	1677,96	0,77	0,22	3,44	0,00057218	0,01303978
501	Gm43360	9,22	-1,92	0,56	-3,44	0,00057606	0,01307769
502	Rpp40	105,75	0,72	0,21	3,44	0,00057892	0,01310923
503	Cyp3a41a	73,41	5,60	1,63	3,44	0,00057922	0,01310923
504	Gm28437	676,19	0,66	0,19	3,43	0,00059934	0,01350891
505	Srp54c	127,35	0,60	0,18	3,43	0,00060715	0,01366619
506	Lama3	326,08	0,62	0,18	3,43	0,00061182	0,01373769
507	H2-Bl	32,30	-1,79	0,52	-3,42	0,0006188	0,01380799

508	Blvrb	1214,04	-0,68	0,20	-3,42	0,00061932	0,01380799
509	Gm38357	21,81	1,88	0,55	3,42	0,00062481	0,01389272
510	BC049987	209,67	0,75	0,22	3,42	0,00062681	0,01389959
511	E030030I06R	24,27	-1,30	0,38	-3,42	0,00063538	0,01405177
512	Sox17	48,92	-0,74	0,22	-3,41	0,00064228	0,01416636
513	Gm15610	12,55	-1,57	0,46	-3,41	0,00064727	0,01422267
514	Arl2bp	102,53	0,67	0,20	3,41	0,00064743	0,01422267
515	Agap2	57,13	-0,86	0,25	-3,41	0,00065431	0,01435461
516	Sh3tc1	118,20	-0,64	0,19	-3,40	0,00066797	0,01459601
517	Gm49201	63,49	0,61	0,18	3,40	0,0006689	0,01459604
518	Ppargc1b	119,04	0,75	0,22	3,40	0,00067058	0,01459604
519	Spata7	29,85	0,84	0,25	3,40	0,00067063	0,01459604
520	Htr1d	5,67	4,28	1,26	3,40	0,0006759	0,01469131
521	Eda2r	6,40	-2,18	0,64	-3,40	0,00068605	0,01487256
522	Gm26621	36,00	-0,84	0,25	-3,38	0,00071452	0,01544898
523	1500004A13I	14,52	1,71	0,51	3,38	0,00071735	0,01546947
524	Gtpbp4-ps1	35,35	1,77	0,52	3,38	0,00071956	0,01547666
525	Zfp791	17,67	1,31	0,39	3,38	0,00073411	0,01574844
526	Cyp2d12	13,83	1,83	0,54	3,37	0,00074616	0,01594737
527	Trmt9b	44,44	0,98	0,29	3,37	0,00074823	0,01594737
528	Gm10222	198,47	0,66	0,20	3,37	0,00075325	0,01603367
529	Slc18a1	21,11	1,33	0,40	3,37	0,00075766	0,0160923
530	Phf13	118,07	-0,64	0,19	-3,37	0,00075796	0,0160923
531	Olfr78	3,88	-3,05	0,91	-3,36	0,00076781	0,01625946
532	Gabbr1	106,38	-0,61	0,18	-3,36	0,0007698	0,01628051
533	Aldh3b2	3,30	4,04	1,20	3,36	0,00077472	0,01636356
534	Lama4	93,27	-0,63	0,19	-3,36	0,00077594	0,01636836
535	Cxcl2	6,90	2,53	0,75	3,36	0,00077951	0,01642259
536	Gm30301	78,22	-0,93	0,28	-3,35	0,00081357	0,01700944
537	Mup16	2274,11	0,72	0,22	3,35	0,00082268	0,0171718
538	Gm9770	7,47	2,88	0,86	3,34	0,0008236	0,0171718
539	Mx1	33,98	0,87	0,26	3,34	0,00082446	0,0171718
540	Pik3r3	59,94	0,80	0,24	3,34	0,00083937	0,01741623
541	Gm37362	7,97	1,73	0,52	3,33	0,0008531	0,01764702
542	Ctse	483,33	1,18	0,35	3,33	0,00085372	0,01764702
543	Mt2	11,64	2,10	0,63	3,33	0,00085665	0,01768545
544	Slc41a1	125,99	0,64	0,19	3,33	0,00085955	0,01772312
545	Ier5	82,08	-0,91	0,27	-3,33	0,00086834	0,01781507
546	Me2	43,72	-0,83	0,25	-3,33	0,00087649	0,01791519
547	mt-Nd3	50,28	0,99	0,30	3,32	0,00088695	0,01808406
548	Ngp	17,19	2,20	0,66	3,32	0,00089242	0,01817318
549	Dmbt1	9,76	2,51	0,76	3,32	0,00089768	0,01821264
550	Lrtm1	367,13	1,41	0,42	3,32	0,00090836	0,01840653
551	Tspan8	15,11	1,53	0,46	3,31	0,00092686	0,01866645
552	Gm14399	15,76	-1,53	0,46	-3,31	0,00094603	0,01893679
553	Gm25047	10,14	-1,92	0,58	-3,30	0,00095073	0,01898452
554	Mab21l3	16,43	-2,15	0,65	-3,30	0,00095638	0,01907427
555	Zfp383	71,10	0,74	0,22	3,30	0,0009604	0,01911311
556	Camta1	91,66	-0,59	0,18	-3,30	0,00097232	0,01929861
557	Pdk4	579,84	-1,48	0,45	-3,30	0,00097536	0,01933558
558	Gm14295	51,69	0,76	0,23	3,29	0,00098695	0,01945193
559	Btbd19	50,85	-1,28	0,39	-3,29	0,00099277	0,01951608
560	Gm25545	6,55	1,92	0,58	3,29	0,00100157	0,01964227
561	Mafb	895,03	1,17	0,36	3,29	0,0010037	0,01966064
562	Col16a1	193,50	2,80	0,85	3,28	0,00102922	0,02006488
563	Otud1	187,00	-0,66	0,20	-3,28	0,00103298	0,02011444
564	Akr1b8	39,13	-0,75	0,23	-3,28	0,00104028	0,02020743

565	Dusp1	1399,29	-0,71	0,22	-3,28	0,00105295	0,02038266
566	mt-Tc	120,84	1,60	0,49	3,27	0,00106276	0,02052425
567	1700017B05I	241,56	-0,70	0,21	-3,27	0,00108346	0,02081062
568	Izumo4	66,36	0,74	0,23	3,27	0,00108391	0,02081062
569	Tbkbp1	116,92	-0,62	0,19	-3,27	0,00109054	0,02091348
570	Gm17025	6,70	1,82	0,56	3,26	0,00109893	0,02102537
571	Pim3	1361,14	1,28	0,39	3,26	0,00112473	0,02144423
572	Adprm	152,31	0,66	0,20	3,26	0,00113216	0,02153586
573	Hcn3	93,50	1,09	0,34	3,25	0,00113949	0,02160038
574	Creld2	1510,29	1,17	0,36	3,25	0,00114565	0,02169223
575	Slc7a15	5,92	2,13	0,66	3,25	0,00115111	0,02177047
576	Cdk1	28,90	-1,00	0,31	-3,25	0,00115645	0,02184639
577	Trib1	732,86	0,84	0,26	3,25	0,00115834	0,02185064
578	Cdkl1	8,76	-1,69	0,52	-3,25	0,00115933	0,02185064
579	Slc26a10	45,94	1,08	0,33	3,24	0,00118411	0,02219056
580	Zfp773	17,71	-1,19	0,37	-3,24	0,00120457	0,02249707
581	Nfxl1	323,91	-0,59	0,18	-3,24	0,00120798	0,02250975
582	Gm4963	19,33	2,02	0,62	3,24	0,00121072	0,02250975
583	Pcdhgb6	4,24	-2,79	0,86	-3,23	0,00123026	0,02284723
584	Corin	363,87	0,81	0,25	3,23	0,00124397	0,02300962
585	Shisa2	3,61	-3,36	1,04	-3,23	0,00125118	0,02310532
586	Fat4	61,52	-0,63	0,20	-3,22	0,00127269	0,02331945
587	Esco2	33,32	-1,21	0,37	-3,22	0,00128534	0,02347083
588	Gm13449	65,54	0,75	0,23	3,22	0,00129458	0,02356309
589	Pitx3	211,12	-2,30	0,71	-3,22	0,00130196	0,02365697
590	Gm16061	7,25	2,50	0,78	3,22	0,00130261	0,02365697
591	Mas1	53,41	0,97	0,30	3,20	0,0013525	0,02442813
592	Gm17767	26,75	1,28	0,40	3,20	0,00136324	0,02459516
593	Pfkfb3	199,61	0,61	0,19	3,20	0,00137357	0,02475431
594	Zfp503	36,95	-0,90	0,28	-3,20	0,00138244	0,02486417
595	Pyroxd1	90,62	0,62	0,19	3,20	0,00138269	0,02486417
596	Rpl7a-ps10	17,14	4,87	1,52	3,20	0,00138826	0,02488284
597	Wfdc12	14,36	4,32	1,35	3,20	0,00139769	0,02497018
598	Wisp1	30,35	-1,11	0,35	-3,19	0,00141646	0,02525077
599	Rims4	345,20	-1,72	0,54	-3,19	0,00143135	0,02546104
600	Gm37795	4,11	-2,74	0,86	-3,19	0,00143951	0,02557848
601	Arhgap10	94,25	1,20	0,38	3,18	0,00145994	0,02585777
602	Igsf3	23,03	-1,02	0,32	-3,18	0,0014671	0,02592871
603	Gm14325	123,35	0,59	0,19	3,18	0,0014752	0,02596679
604	Col15a1	182,06	-0,65	0,20	-3,18	0,00147556	0,02596679
605	Ccnb2	18,91	-1,36	0,43	-3,18	0,00149717	0,0262627
606	Abcb1a	66,23	0,87	0,27	3,17	0,00149904	0,02626749
607	Ankrd55	56,00	-0,75	0,24	-3,17	0,00151602	0,02650862
608	Slc9b2	3,16	3,98	1,26	3,17	0,00152731	0,02664942
609	Cyp3a11	106684,27	-1,63	0,51	-3,17	0,00153137	0,02669196
610	Exoc3l	55,91	-0,62	0,20	-3,16	0,001552	0,02698139
611	Sox18	95,89	-0,68	0,21	-3,16	0,0015529	0,02698139
612	Cdc20	35,89	-2,24	0,71	-3,16	0,00157741	0,02732073
613	Arl4a	427,99	-0,73	0,23	-3,16	0,0015964	0,02757475
614	Gbp6	41,22	-0,77	0,25	-3,15	0,0016304	0,02805523
615	Mbd4	57,05	-0,62	0,20	-3,15	0,00165084	0,02832362
616	Iqgap3	10,90	-1,71	0,54	-3,14	0,00166555	0,02851711
617	Creb3l2	530,45	-0,73	0,23	-3,14	0,00167347	0,02858483
618	Erp27	39,06	1,00	0,32	3,14	0,00167708	0,02859543
619	Etfb	274,94	-0,60	0,19	-3,14	0,00168271	0,02866001
620	B230206H07	38,28	-0,94	0,30	-3,14	0,00168435	0,02866001
621	Gm15344	53,51	-0,96	0,31	-3,14	0,00168895	0,02868322

622	mt-Te	118,50	1,74	0,55	3,14	0,00169523	0,02872642
623	Sox12	103,25	-0,61	0,19	-3,14	0,00170587	0,02884722
624	Nol4l	36,61	-0,80	0,26	-3,13	0,00176186	0,02955112
625	Fam129b	119,90	-0,65	0,21	-3,13	0,00176784	0,02960051
626	Hspa5	23709,03	0,63	0,20	3,13	0,0017684	0,02960051
627	Rpl29	720,29	1,10	0,35	3,13	0,00177283	0,02962317
628	Kif26a	38,85	-0,82	0,26	-3,13	0,00177574	0,02962317
629	Hhipl1	19,64	1,28	0,41	3,13	0,00177696	0,02962317
630	Gm26608	49,62	0,80	0,26	3,12	0,00178103	0,02964865
631	Gm26782	16,23	1,15	0,37	3,12	0,00179691	0,02977483
632	Wif1	4,56	3,93	1,26	3,12	0,00182082	0,03004431
633	Gm5884	10,53	-1,47	0,47	-3,12	0,00182784	0,03004525
634	Gm15337	17,37	1,62	0,52	3,12	0,00183073	0,03006271
635	Ppp1r14a	267,45	0,59	0,19	3,11	0,00184926	0,03023007
636	Gm4366	370,28	0,62	0,20	3,11	0,00186107	0,03037905
637	E2f2	59,10	0,60	0,19	3,11	0,0018688	0,03047498
638	Gvin1	4,61	-2,87	0,92	-3,11	0,00187391	0,0305281
639	Stx3	105,10	-0,63	0,20	-3,11	0,00188818	0,03065742
640	5033406O09	18,11	-3,06	0,98	-3,11	0,0018893	0,03065742
641	Gm37255	3,76	3,20	1,03	3,10	0,00191904	0,0309185
642	Slc16a14	2,98	4,52	1,46	3,09	0,00196843	0,03162942
643	Kif20a	23,04	-1,24	0,40	-3,09	0,00197479	0,03170068
644	Cldn18	4,15	2,99	0,97	3,09	0,00199995	0,03191774
645	Gm10175	10,78	1,52	0,49	3,09	0,00201254	0,03205379
646	Col6a2	143,96	-0,63	0,20	-3,09	0,00201432	0,03205379
647	Col12a1	54,21	-0,91	0,29	-3,09	0,00203339	0,03223255
648	5330417C22R	39,49	1,10	0,36	3,08	0,00204881	0,03244521
649	Gm34667	106,73	0,65	0,21	3,08	0,00206293	0,03254399
650	Zc3h6	50,47	0,92	0,30	3,08	0,00206519	0,03254856
651	Ppp1r13l	67,00	-0,67	0,22	-3,08	0,00207762	0,03268644
652	Adra2c	8,61	5,49	1,78	3,08	0,00208396	0,03275021
653	Rapgefl1	23,78	1,14	0,37	3,08	0,00209035	0,0328069
654	Kcnj10	42,11	-1,04	0,34	-3,07	0,00211598	0,03312686
655	Gm45674	50,45	0,81	0,26	3,07	0,00213773	0,03337279
656	Eid1	114,01	0,69	0,23	3,07	0,0021474	0,03345954
657	Trim34b	48,86	0,65	0,21	3,06	0,00219738	0,03410908
658	Gpm6a	41,30	0,81	0,27	3,06	0,00220279	0,03412877
659	Ppbp	9,12	2,17	0,71	3,05	0,00225326	0,03476642
660	Gm40787	18,13	1,37	0,45	3,05	0,00226774	0,03488385
661	Ptp4a3	132,03	-0,61	0,20	-3,05	0,00226906	0,03488385
662	Gm35190	8,23	-1,83	0,60	-3,05	0,00229005	0,03514966
663	Mup-ps11	4,57	2,38	0,78	3,05	0,00232009	0,03544908
664	Ccl24	42,02	-0,81	0,27	-3,05	0,00232033	0,03544908
665	Evc2	18,28	2,08	0,68	3,05	0,00232256	0,03545035
666	Hmmr	15,64	-1,76	0,58	-3,04	0,0023481	0,03574065
667	Gm37131	11,03	1,40	0,46	3,04	0,00235891	0,03583892
668	Aldh1a7	2422,66	-0,72	0,24	-3,04	0,00236612	0,03591528
669	Renbp	44,20	-0,87	0,29	-3,04	0,00237353	0,03599455
670	B4galnt3	18,54	1,50	0,49	3,04	0,00237746	0,03602094
671	Ttc39a	140,38	0,62	0,20	3,04	0,00240234	0,03617865
672	Elane	4,16	3,35	1,11	3,04	0,00240326	0,03617865
673	Zfp941	17,89	1,29	0,43	3,03	0,00242404	0,03638767
674	Hsd3b1	36,45	1,15	0,38	3,03	0,0024282	0,03638767
675	Gnat2	42,55	0,87	0,29	3,03	0,00244668	0,03663115
676	Atp13a5	4,30	3,39	1,12	3,03	0,0024751	0,03689331
677	Ace2	14,08	1,30	0,43	3,03	0,0024754	0,03689331
678	Cbfa2t3	63,82	-0,66	0,22	-3,03	0,0024853	0,03700292

679	Gm44421	44,93	1,00	0,33	3,02	0,00248725	0,03700292
680	Cntnap1	50,72	0,74	0,25	3,02	0,00249766	0,03712415
681	Neto2	16,04	-1,14	0,38	-3,02	0,00252941	0,03750732
682	Mgst3	150,73	-0,89	0,29	-3,02	0,00253027	0,03750732
683	Gm42559	7,90	-1,63	0,54	-3,02	0,00256971	0,03795505
684	Gm16559	96,41	0,83	0,28	3,01	0,00257806	0,03802858
685	Ggta1	61,25	-0,64	0,21	-3,01	0,00257931	0,03802858
686	Gm42047	613,98	-1,18	0,39	-3,01	0,00264647	0,03894896
687	Lmod3	3,73	3,62	1,21	3,00	0,0026905	0,03942076
688	Gm11361	227,44	-0,89	0,30	-3,00	0,0027203	0,03978358
689	Plet1	40,06	1,19	0,40	2,99	0,00276674	0,04025104
690	Rad54b	28,89	-0,88	0,29	-2,99	0,00278637	0,04050086
691	C030037D09I	43,67	-0,94	0,32	-2,99	0,00279416	0,04057793
692	Hkdc1	9,14	2,39	0,80	2,99	0,00280181	0,04061756
693	Mki67	67,77	-1,01	0,34	-2,99	0,0028296	0,04092532
694	Crybb3	29,60	1,37	0,46	2,98	0,00284716	0,04113011
695	Gm29650	22,64	-0,93	0,31	-2,98	0,00288252	0,04145232
696	Gm14400	3,84	2,59	0,87	2,98	0,00288458	0,04145232
697	Gm13835	32,74	1,22	0,41	2,97	0,00293572	0,04200384
698	Pde6h	17,57	0,99	0,33	2,97	0,00297204	0,04241279
699	Atp8a1	113,56	-0,73	0,25	-2,97	0,00298892	0,04261673
700	Fam78a	29,48	-0,92	0,31	-2,97	0,00300063	0,04270971
701	5430402O13	11,39	1,39	0,47	2,97	0,00301714	0,04283344
702	Prtn3	89,84	3,10	1,05	2,96	0,00307473	0,04353845
703	Rgs1	90,70	1,69	0,57	2,95	0,00313257	0,04420527
704	Them6	61,36	-0,65	0,22	-2,95	0,00315755	0,04444344
705	Gm45738	4,41	2,55	0,87	2,95	0,00317021	0,04454544
706	Epb41l1	53,48	-0,74	0,25	-2,95	0,0032128	0,04506688
707	Gm32391	30,54	0,89	0,30	2,94	0,003234	0,04524878
708	Dvl2	101,26	-0,64	0,22	-2,94	0,00323544	0,04524878
709	Tmem158	12,05	-1,24	0,42	-2,94	0,00323677	0,04524878
710	Kif22	19,90	-1,11	0,38	-2,94	0,00327617	0,04549037
711	Gstm4	982,41	-0,68	0,23	-2,94	0,00332207	0,04604995
712	mt-Nd6	12582,57	1,17	0,40	2,93	0,00334217	0,04617302
713	Havcr1	27,32	3,24	1,11	2,93	0,00336388	0,04639507
714	Prr15l	8,30	1,68	0,58	2,93	0,00339328	0,04664416
715	Ildr2	37,75	-1,52	0,52	-2,93	0,0034133	0,04676304
716	Knl1	9,74	-1,47	0,50	-2,92	0,00345707	0,04713948
717	Gm15540	15,70	-1,24	0,43	-2,92	0,00345791	0,04713948
718	Gpr65	17,72	1,15	0,39	2,92	0,0034616	0,0471424
719	B930025P03I	21,85	1,16	0,40	2,92	0,00346525	0,0471424
720	Cfb	80,65	-0,74	0,25	-2,92	0,00349791	0,04734701
721	Zscan20	57,24	-0,59	0,20	-2,92	0,00349884	0,04734701
722	Abcd2	68,88	-1,08	0,37	-2,92	0,00350714	0,04737801
723	Tnfrsf25	26,65	-2,60	0,89	-2,91	0,00358743	0,04823865
724	Gm8615	142,78	-0,64	0,22	-2,91	0,00358843	0,04823865
725	Cnn1	3,33	3,00	1,03	2,91	0,00360471	0,04833496
726	Adamts6	37,69	0,77	0,26	2,91	0,00360735	0,04833496
727	Gm48342	7,55	-1,98	0,68	-2,91	0,00366571	0,04891774
728	Agbl3	74,49	0,61	0,21	2,90	0,00374821	0,04973637
